# Immunological markers of *Plasmodium vivax* exposure and immunity: a systematic review and meta-analysis

**DOI:** 10.1186/s12916-014-0150-1

**Published:** 2014-09-09

**Authors:** Julia C Cutts, Rosanna Powell, Paul A Agius, James G Beeson, Julie A Simpson, Freya J I Fowkes

**Affiliations:** Macfarlane Burnet Institute of Medical Research, 85 Commercial Road, Melbourne, VIC 3004 Australia; Department of Microbiology, Monash University, Melbourne, Victoria Australia; Department of Medicine, University of Melbourne, Victoria, Australia; Centre for Epidemiology and Biostatistics, University of Melbourne, Melbourne, Australia; Department of Epidemiology and Preventive Medicine and Department of Infectious Diseases, Monash University, Melbourne, Australia

**Keywords:** Malaria, *Plasmodium vivax*, Immunity, Antibodies, Epidemiology, Systematic review, Meta-analysis

## Abstract

**Background:**

Identifying *Plasmodium vivax* antigen-specific antibodies associated with *P. vivax* infection and protective immunity is key to the development of serosurveillance tools and vaccines for malaria. Antibody targets of *P. vivax* can be identified by seroepidemiological studies of individuals living in *P. vivax*-endemic areas, and is an important strategy given the limited ability to culture *P. vivax in vitro*. There have been numerous studies investigating the association between *P. vivax* antibody responses and *P. vivax* infection, but there has been no standardization of results to enable comparisons across populations.

**Methods:**

We performed a systematic review with meta-analysis of population-based, cross-sectional, case–control, and cohort studies of individuals living in *P. vivax*-endemic areas. We searched 6 databases and identified 18 studies that met predefined inclusion and quality criteria, and examined the association between antibody responses to *P. vivax* antigens and *P. vivax* malaria.

**Results:**

The majority of studies were published in South America (all from Brazil) and the rest from geographically diverse areas in the Asia-Pacific region. Considerable heterogeneity in estimates was observed, but IgG responses to *Pv*CSP, *Pv*MSP-1_19_, *Pv*MSP-9_RIRII_, and *Pv*AMA1 were associated with increased odds of *P. vivax* infection in geographically diverse populations. Potential sources of heterogeneity included study design, different transmission intensities and transmigrant populations. Protective associations were observed for antibodies to *Pv*MSP-1_19_, *Pv*MSP-1_NT_, *Pv*MSP-3α and *Pv*MSP-9_NT_ antigens, but only in single geographical locations.

**Conclusions:**

This systematic review revealed several antigen-specific antibodies that were associated with active infection and protective immunity, which may be useful biomarkers. However, more studies are needed on additional antigens, particularly cohort studies to increase the body of evidence for protective immunity. More studies representing diverse geographical regions encompassing varying *P. vivax* endemicities are needed to validate the generalizability of the findings and to provide a solid evidence base for the use of *P. vivax* antigens in vaccines and serosurveillance tools.

**Electronic supplementary material:**

The online version of this article (doi:10.1186/s12916-014-0150-1) contains supplementary material, which is available to authorized users.

## Background

*Plasmodium vivax* is the most widely distributed species of human malaria, with an estimated 40% of the world’s population being at risk of *P. vivax* infection [1]. The majority of *P. vivax* infections occur in Central and South-East Asia, and there are approximately 80 to 300 million clinical cases of *P. vivax* malaria each year [1]. Despite the large burden of disease, *P. vivax* has traditionally been neglected because it has been considered a relatively benign form of malaria. Now it is recognized that *P. vivax* can cause severe disease (similar to that of *Plasmodium falciparum*) [2] and, together with increased recognition of the burden, there are renewed efforts in the development of *P. vivax*-specific interventions (that is, vaccines) and surveillance tools (diagnostics, serosurveillance) to expedite the goal of malaria elimination and eradication [3,4].

Currently, only two *P. vivax* vaccine candidates (*Pv* Duffy binding protein (*Pv*DBP) and *Pv* circumsporozoite protein *Pv*CSP) are in clinical trials (Phase I) compared with 23 *P. falciparum* vaccine candidates (including one in Phase III trials: RTS,S) [5,6]. This may reflect the previous neglect of *P. vivax*, the difficulty in maintaining *P. vivax* in culture, and the limited animal models of infection currently available. Such technical challenges have hindered the ability to prioritize *P. vivax* candidate antigens against pre-clinical selection criteria, including knowledge of protein function and antigenic diversity, and demonstrations that antibodies against an antigen inhibit growth *in vitro* or function in other ways, or are protective in animal models of infection [7]. In the absence of an *in vitro* system, *P. vivax* antigens can be selected based on *P. falciparum* homologues and an additional pre-clinical selection criterion, namely, that the antigen induces naturally acquired immunity in individuals living in malaria-endemic areas [7].

In *P. vivax*-endemic areas, the prevalence and density of *P. vivax* infection and the incidence of *P. vivax* symptomatic malaria decrease with age [8]. This epidemiological pattern reflects the acquisition of natural immunity that develops after repeated exposure [9]. This immunity is non-sterilizing and does not protect against infection, but acts by reducing parasite numbers in the blood and the subsequent clinical symptoms. Antibodies are thought to be an important component of naturally acquired immunity, and are considered to be biomarkers of both immunity and exposure. Potential antibody targets include *P. vivax* antigens expressed on sporozoites (the pre-erythrocytic liver stage), the invading merozoite and the surface of infected erythrocytes (erythrocytic stage) and the gametocyte (sexual stage) [8]. *P. vivax* also has an additional dormant stage in the liver, the hypnozoite, which is believed to be responsible for relapses in *P. vivax* infection [10].

There have been numerous studies investigating associations between *P. vivax* immune responses and *P. vivax* infection, but there is considerable heterogeneity between studies, both in terms of methodology and presentation of results, making cross-study comparison problematic. Here, we aimed to review and synthesize the literature, by standardizing analyses and identifying targets of naturally acquired immunity to *P. vivax*, which we have previously done similarly for *P. falciparum* [11]. There were two key objectives of this study: to determine antigen-specific antibody responses associated with infection, and to determine antibody responses associated with protective immunity. We included cross-sectional and case–control studies in order to identify markers of *P. vivax* infection, and also included cohort studies, which provide the highest level of evidence to detect causal effect in observational research, in order to identify antibody responses that protect against *P. vivax* malaria. The overarching aim of the study was to provide a more comprehensive understanding of antibody-mediated immunity to *P. vivax* and, more specifically, to help inform the development of vaccines and serosurveillance tools to facilitate the control, elimination and eradication of *P. vivax*.

## Methods

The Meta-analysis of Observational Studies in Epidemiology (MOOSE) working group [12] guidelines and the Preferred Reporting Items for Systematic Reviews and Meta-Analyses (PRISMA) specifications were adhered to in the conducting and reporting of this systematic review and meta-analysis [13]. For a completed PRISMA checklist, see Additional file 1.

### Search methods for identification of studies

PubMed, Web of Science, Scopus, Google Scholar, African Index Medicus, and the Latin American and Caribbean Health Sciences Literature (LILACS) databases were searched for studies published in all years up to and including 30 November 2013 that examined the association of antibody responses to *P. vivax* antigens with *P. vivax* infection or *P. vivax* malaria. Key words included: malaria, vivax, plasmodium, immunoglobulin, IgG, antibody, immunity, rhoptry, microneme, sporozoite, CSP, circumsporozoite, TRAP, thrombospondin, merozoite, MSP, AMA, DBP, Duffy binding protein, EBA, EBP, erythrocyte binding*, EMP, erythrocyte membrane protein, RBL, reticulocyte binding like protein, RBP, reticulocyte binding protein, VSA, variant surface antigen, VIR, gameotocyte, transmission blocking, Pvs25, ookinete surface protein, Pvs28, sexual stage surface protein, transmission-blocking target antigen, and Pvs230. The full search strategy for one database (PubMed) is provided (see Additional file 2). The reference lists of the obtained papers were searched for further studies. Studies reported in languages other than English were included, and were translated into English using online translation applications. *A priori*, we did not formally attempt to identify unpublished population studies because this would have required us to provide substantial descriptions of the study design, sample testing, and analysis used in the studies, and a review of ethical and other issues.

### Criteria for considering studies

#### Study designs and study participants

Population-based cross-sectional, case–control, and cohort studies, including treatment to re-infection studies, were included in the systematic review. Randomized controlled trials and vaccine efficacy trials of blood-stage vaccines were excluded because rigorous inclusion and exclusion criteria are applied in these studies, thus the participants are, typically, not representative of the general population. The primary criterion for study inclusion was inclusion of individuals (children, adults, and/or pregnant women) living in *P. vivax*-endemic areas. All geographical locations were included. Studies of the following types of populations were excluded because they were unlikely to represent the general population: populations experiencing epidemic malaria (that is, no previous exposure to *P. vivax*); returned travellers; military personnel; and populations in which greater than 20% of individuals were transmigrants who had resided in the area for less than 5 years at the time of sampling. Studies that included multiple population subsets were assessed on a sub-population basis to determine eligibility for inclusion.

#### Antibody measures

Studies that measured total immunoglobulin G (IgG), total IgM, or IgG subclass (1–4) responses to recombinant or synthetic defined *P. vivax* antigens were considered. Antibody responses to full-length proteins, processing products, and defined regions of *P. vivax* antigens from any life-cycle stage and any subcellular location were also included. Responses to peptides representing undefined regions or incomplete domains or subdomains of antigens were excluded, with the exception of proteins or defined domains that could not be expressed as a single product. In such cases, responses to a combination of protein fragments representing the full-length protein or domain were analyzed. For cohort studies, if antibody responses were measured at multiple time points the baseline (that is, time 0), antibody responses were analyzed. Data from cohort studies in which antibody responses were determined after malariometric measures were excluded.

#### Malaria outcome measures

The following malaria outcome measures were included: *P. vivax* infection, high-density *P. vivax* infection, and symptomatic *P. vivax* malaria, using the definitions as described in the individual studies. In cohort studies, *P. vivax* re-infection was also included as an outcome. Studies in which malariometric measures were determined retrospectively (for example, where cumulative history of malaria exposure was the exposure or outcome of interest) were excluded.

#### Quality criteria

The minimum quality criteria for inclusion were: confirmation of *P. vivax* parasitemia by light microscopy, rapid detection kit, or PCR; detection of *P. vivax* malaria by active and/or passive case detection; and symptomatic malaria defined by fever and/or history of fever (within the past 72 hours) plus *P. vivax* parasitemia. In studies in which symptomatic malaria was the outcome of interest, cases of symptomatic malaria in individuals with *P. falciparum* and *P. vivax* co-infection were excluded because the symptoms could not be attributed exclusively to one or the other species. Cut-offs for positive antibody responses by ELISA had to be defined by the use of unexposed (malaria-naïve) controls rather than individuals from the same exposed population found to be *P. vivax*-negative at the time of sampling. For treatment to re-infection studies, if treatment failure was accounted for, it had to be defined by either genetic analysis or documented clearance of infection within a specified time frame appropriate for the chosen antimalarial. In case–control studies, at least one control for every case had to be recruited from the same population (that is, studies that recruited a small number of laboratory controls for antibody comparison purposes were excluded).

### Selection of studies

Two independent review authors used the inclusion and exclusion criteria to screen titles and abstracts. The full text of potentially relevant studies was retrieved and examined for compliance with the inclusion and exclusion criteria by the same two review authors independently. Discrepancies were resolved by discussion with a third author.

#### Effort to include all available studies and data

Authors of original studies were contacted if relevant information on the study population, eligibility criteria, or key study data were not presented in the published report. For studies in which antibody responses to *P. vivax* antigens were described, but no details of *P. vivax* outcomes were reported, authors were invited to provide malariometric data to enable the study to be included in the review. If authors were unable to provide estimates or data, the study was classified as not meeting the inclusion and/or quality criteria, and was excluded from the systematic review. Cross-sectional data from cohort studies was extracted for inclusion in cross-sectional analyses. In studies in which multiple cross-sectional surveys were performed in the same population, estimates were reported for individual surveys if the data were available. For studies in which antibody responses were analysed as the outcome variable, data were re-analyzed so that malaria or *P. vivax* infection was the outcome variable.

### Risk of bias in individual studies

At an individual study level, selection bias was assessed to determine whether participants were representative of the general population by reviewing individual study inclusion and exclusion criteria. Selection bias in case–control studies was assessed by assessing the comparability of cases and controls as part of the systematic review quality criteria. We excluded case–control studies if the source population differed between cases and controls, because bias would be introduced into the estimates of the association between *P. vivax* antibodies and outcomes. An additional selection bias can occur in case–control studies when cases and/or controls are selected based on criteria relating to their exposure (that is, antibody) status or when there are differences in the reporting of exposure between cases and controls. However, this is unlikely because immunoassays would be performed after enrolment into the study. Information bias (resulting from flaws in measuring antibody and *P. vivax* outcome data) are unlikely because antibodies are measured using immunoassays that are standardized within each study and across outcome groups. The quality criterion of this review ensured accurate measurement of *P. vivax* outcomes, and it is unlikely that measurement of outcomes would differ depending on antibody groups. To reduce bias further, we excluded studies that measured the *P. vivax* outcome prior to antibody determination. Although these studies may be useful in determining markers of exposure, we excluded such studies because unmeasured *P. vivax* exposure and/or *P. vivax* antibody decay between measurements may lead to misclassification and bias in estimates of association. For measures of association, estimates adjusted for demographic variables and/or spatial confounders are reported where possible to reduce confounding. Estimates adjusted for other anti-*P. vivax* antibodies are not reported because antibody responses are typically highly correlated, making it difficult to estimate their individual regression coefficients reliably [11]; in these cases unadjusted estimates are reported.

### Data analysis

#### Data collection

Measures of association (odds ratio (OR), risk ratio (RRs), incidence rate ratio (IRR), or hazard ratios (HR)) and their 95% confidence intervals (CIs) were extracted or derived using data reported in the publications. Data extraction was performed independently by two reviewers using a proforma. Contact with authors was established through an initial email explaining the nature of the systematic review and the information required, together with the proforma. If the corresponding author did not respond within three email attempts, no further action was taken. Where a study did not provide measures of association (or they could not be calculated using the information provided) the study results were used only for qualitative analysis.

#### Standardization of antibody measures

Measurement of antibody levels by ELISA does not produce a common metric between studies. Individuals can be classified as ‘responders’ or ‘non-responders’ relative to a negative control (unexposed sera) within each study. Study-specific comparisons of these exposure variables can then be pooled [11]. However, categories based upon arbitrary cut-offs (including categories of responders based on statistical rankings) cannot be pooled across studies. For studies in which the antibody measures were analyzed as continuous exposure variables, authors were asked to reanalyze their data to provide a binary classification of responders versus non-responders.

#### Standardization of malaria outcome measures

Studies were grouped according to the study design used to examine the relationship between *P. vivax* antibodies and *P. vivax* outcome. For cross-sectional and case–control studies, OR was extracted or calculated, and for cohort studies, RR, HR, and IRR were extracted or calculated where possible, or unadjusted ORs were converted to RR [14] (RR, HR, and IRR are hereinafter denoted as RR). An RR/OR of 1 indicates that the risk/odds of malaria is equal for those with (responders) and those without (non-responders) antibody responses. Separate estimates were obtained for *P. vivax* detected by light microscopy, PCR, and ligase detection reaction–fluorescent microsphere assay (LDR-FMA). For meta-analyses, estimates using different parasite detection methods were combined. For studies in which multiple estimates were reported for different detection methods, the estimate reflecting the more commonly used method was presented in the forest plot to enable comparisons, and other estimates were presented in the text. Where zero counts were present in 2 × 2 tables, a constant value (0.5) was added to all cells to enable estimation of the OR/RR.

Our aim was to obtain a single estimate for each antibody response and *P. vivax* outcome. If antibody responses to the same antigen in the same population-based study were reported in several publications, results from the largest sample size were used. Separate estimates were obtained for the OR/RR associated with the *Pv*CSP repetitive domain (VK210, VK247, *P. vivax*-like alleles, NS1/81-V20 antigen, and VK210 and VK247 chimeric antigen), *Pv* merozoite surface protein (*Pv*MSP)-1_19_, *Pv*MSP-1 N-terminus, *Pv* apical membrane antigen (P*v*AMA1) ectodomain, *Pv*DBP (DBP region II AH, O, P, and Sal 1 alleles, DBP regions II-IV, Sal 1 allele), *Pv*MSP-9 (Block I and Block II repeats, Block II repeats, N-terminal region), *Pv*MSP-3α (full-length, N-terminal region, Block I repeats, Block II repeats), *Pv*MSP-5, *P. vivax* reticulocyte binding protein (PvRBP1; the extracellular domain was expressed as overlapping recombinant fragments), and *Pv*SERA4. Together with 95% CIs and *P* values, we interpreted a 20% relative difference in odds/risk of *P. vivax* outcome to be a clinically meaningful difference between antibody groups *a priori*, which is approximately half of the observed efficacy of the *P. falciparum* RTS,S vaccine in Phase III trials [15,16].

#### Synthesis of results: meta-analysis

A meta-analysis was performed for each antigen, stratified by study design and *P. vivax* outcome. Where there were two or more studies that could be combined, a pooled estimate for each outcome was calculated using either a fixed-effects or random-effects model. The standard error of the natural logarithm (ln) of the RR/OR was calculated using the formula: (ln(upper limit of CI) –ln(estimate))/1.96. For fixed-effects models, pooled effects estimates were weighted by the inverse of the individual study standard error. Where random-effects models were specified, a between-study variance component was incorporated into the study weights [17]. Between-study heterogeneity was measured with the *I*^2^ statistic, and represents the percentage of variation in a pooled estimate attributable to between-study variability [18]. Tests for significant between-study heterogeneity were also reported, and were based on the weighted sum of the differences between study estimates and the overall pooled estimate; the statistic takes a χ^2^ distribution with degrees of freedom equal to the number of studies minus 1 [17]. If heterogeneity was 30% or less, a meta-analysis based on a fixed-effects model was specified; otherwise a random-effects approach was used. Where the heterogeneity exceeded 75% and/or the heterogeneity test statistic was significant at *P* < 0.1, a pooled effect was not estimated [18-21]. Owing to the small number of studies included in the meta-analyses, sensitivity analyses and assessments of publication bias were not performed. All analyses were performed using STATA software (V11; StataCorp, College Station, TX, USA).

## Results

### Identification and description of included studies

**Figure 1:**
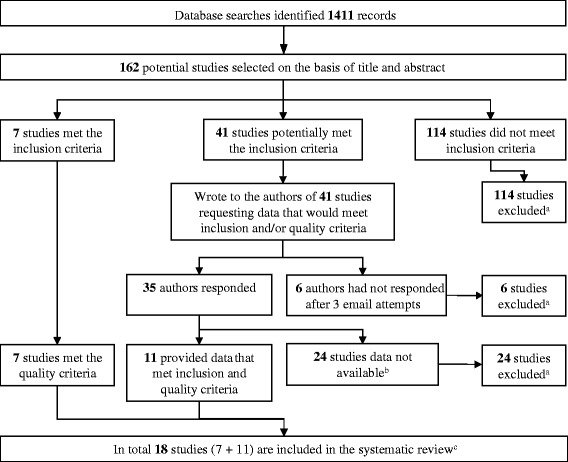
**Flow chart of study identification.** ^**a**^For details of excluded studies, see Additional file 3. ^**b**^Data not in format for re-analysis or data not available. ^**c**^The characteristics of the included studies are given in Table 1.

The database searches identified 1,411 records, of which 162 potentially relevant studies were identified, based upon title and abstract. The full texts of these 162 studies were examined to determine whether they complied with eligibility criteria: 114 did not meet the inclusion criteria (see Additional file 3), 7 fulfilled the inclusion and quality criteria (Figure 1), and 41 studies potentially met inclusion and quality criteria. The authors of the 41 studies that potentially met inclusion and quality criteria were contacted, yielding a further 11 studies that met inclusion and quality criteria, providing a total of 18 studies that were included in the review [22-39] (Figure 1). Details of these 18 studies are shown in Table 1. Of these 18 studies, 9 were cross-sectional, 6 were cohort (4 of which also provided cross-sectional data), and 3 were case–control studies. One cohort study contributed two publications [34,35], and one publication provided data from two countries [22]. For the purpose of this review, we shall refer to each publication as a study.

**Table 1:** **Characteristics of studies included in the systematic review by country**

**Author, year [reference]**	**Region**	**Age range, years**	**Antibody response (type)**	**Study design (n)** ^**a**^	***Plasmodium vivax*** **outcome** ^**b**^
**Brazil**					
Fernandez-Becerra, 2010 [22]^c^	Rio Machado	DNS	*Pv*MSP-1_NT_, *Pv*MSP-1_19_ (IgG)	CS (87)	*Pv* infection (LM or PCR), symptomatic *Pv*
Kano, 2012 [23]	Presidente Figueiredo, Amazonas	9 to 44	*Pv*DBPII-IV, *Pv*MSP-1_19_ (IgG)	CS (432)	*Pv* infection (LM or PCR), symptomatic *Pv*
Lima-Junior, 2008 [24]^d^	Rondonia	10 to 85	*Pv*MSP-9_RIRII_, *Pv*MSP-9_RII_, *Pv*MSP-9_NT_ (IgG)	CS (282)	*Pv* infection
Lima-Junior, 2011 [25]	Rondonia	10 to 81	*Pv*MSP-3α_FL_, *Pv*MSP-3α_NT_, *Pv*MSP-3α_RI_, *Pv*MSP-3α_RII_, *Pv*MSP-3α_CT_ (IgG)	CS (282)	*Pv* infection
Lima-Junior, 2012 [26]	Rondonia	11 to 89	*Pv*MSP-1_19_ (IgG)	CS (277)	*Pv* infection
Nogueira, 2006 [27]	Portuchuelo, Rondonia	DNS	*Pv*MSP-1_NT_, *Pv*MSP-1_19_ (IgG)	Cohort (173)	*Pv* infection
Oliveira-Ferreira, 2004 [28]	Candeias do Jamari, Rondonia	12 to 74	*Pv*CSP (VK210, VK247, *P. vivax*-like) (IgG)	CS (61)	*Pv* infection
Souza-Silva, 2010 [29]	Acre	5 to 90	*Pv*DBPII-IV (IgG)	Cohort (CS)^e^ (366)	*Pv* infection, *Pv* infection (LM or PCR)
Tran, 2005 [30]^f^	Colina and Ribeirinha, Rondonia	11 to 75	*Pv*RBP1 (IgG)	CS (87)	*Pv* infection
Versiani, 2013 [31]	Rio Pardo	DNS	*Pv*MSP-1_NT_ (IgG, IgG1, IgG2, IgG3, IgG4)	Cohort (CS)^e^ (308)	*Pv* infection, symptomatic *Pv*
I**ndonesia**					
Ak, 1998 [32]	Robek	0 to 73	*Pv*MSP-1_19_ (IgG + IgM)	CS (169)	*Pv* infection
Woodberry, 2008 [33]	Timika, Papua	3 to 60	*Pv*MSP-5 (IgG, IgM, IgG1, IgG2, IgG3, IgG4)	CC (340)	Symptomatic *Pv*
Papua New Guinea					
Cole-Tobian, 2009 [34]	Madang	5 to 14	*Pv*DBPII (AH, O, P, Sal 1), *Pv*MSP-1_19_ (IgG)	Cohort^g^ (206)	*Pv* infection, *Pv* infection (LDR-FMA), *Pv* infection >150 parasites/μl
Fernandez-Becerra, 2010 [22]	Madang	0.25 to 3	*Pv*MSP-1_NT_, *Pv*MSP-1_19_ (IgG)	CS (100)	*Pv* infection (LM or PCR), symptomatic *Pv*
King, 2008 [35]	Madang	5 to 14	*Pv*DBPII binding inhibitory antibodies	Cohort (206)^h^	*Pv* infection
Stanisic, 2013 [36]	East Sepik	0.9 to 3.1	*Pv*MSP-3α_NT_, *Pv*MSP-3α_RI_, *Pv*MSP-3α_RII_, *Pv*MSP-3α_CT_, *Pv*MSP-9_NT_, *Pv*MSP-9_RIRII_ (IgG)	Cohort (CS)^e^ (183)	*Pv* infection (LDR-FMA), symptomatic *Pv*
**Thailand**					
Fowkes, 2012 [37]	Mae Sot, Tak	15 to 42^i^	*Pv*AMA1-ecto (IgG)	Nested CC (467)	*Pv* infection
Wongsrichanalai, 1991 [38]	Chanthaburi	DNS	*Pv*CSP (VK210) (IgG)	CC (126)	*Pv* infection
**Turkey**					
Yildiz Zeyrek, 2011 [39]	Sanliurfa	0 to 77	*Pv*MSP-1_19_, *Pv*AMA1-ecto, *Pv*SERA4, *Pv*CSP (VK210 and VK247 chimera) (IgG, IgM, IgG1, IgG2, IgG3, IgG4)	CS (195)	*Pv* infection

CC, case–control; CS, cross-sectional; DNS, did not state; LDR-FMA, ligase detection reaction–fluorescent microsphere assay; LM, light microscopy; *PV*, *Plasmodium vivax*.

^a^Sample size refers to number of participants for whom serology was determined.

^b^ *P. vivax* infection was determined by light microscopy unless otherwise stated.

^c^Fernandez-Becerra, 2010 [22] reported studies performed in two countries and features twice in Table 1.

^d^The studies described by Lima-Junior in 2008 and 2012 [24,26] were conducted in the same area, but the participants were different.

^e^Cohort study with cross-sectional data also included.

^f^Tran, 2005 [30] included data from two different study sites in Brazil.

^g^Treatment to re-infection study.

^h^King, 2008 [35] reported estimates from the same treatment to re-infection study as that described by Cole-Tobian, 2009 [34].

^i^This study comprised pregnant women.

The included studies reported data from Brazil (n = 10), Papua New Guinea (n = 4), Indonesia (n = 2), Thailand (n = 2), and Turkey (n = 1) (Table 1). Sample sizes of the included studies ranged from 61 to 432 study participants, and the majority of studies included both children and adults (4 included children only, 1 included pregnant women only). Antibody responses to *P. vivax* erythrocytic stage proteins were the main antigens studied (*Pv*MSP-1, n = 8; *Pv*DBP, n = 4; *Pv*MSP-3α, n = 2; *Pv*MSP-5, n = 1; *Pv*MSP-9, n = 2; *Pv*AMA1, n = 2; *Pv*RBP1, n = 1; *Pv*SERA4, n = 1) and three studies examined antibody responses to the pre-erythrocytic stage protein *Pv*CSP (Table 1). No studies examined responses to gametocyte antigens. Details of the recombinant antigens investigated are outlined in detail (see Additional file 4). Total IgG responses were analyzed in 17 studies, with IgM and IgG subclass responses being examined in 4 studies each (Table 1). IgG1 and IgG3 subclasses were the predominant IgG subclass responses in all studies [27,31,33,39,40] (see Additional file 5). *P. vivax* infection was the most commonly examined outcome (n = 18), followed by symptomatic *P. vivax* infection (n = 5) and high-density *P. vivax* infection (>150 parasites/μl, n = 1). Light microscopy was used for *P. vivax* detection in the majority of studies (n = 13), with four studies using PCR or microscopy and three using LDR-FMA. For the purpose of the review, all *P. vivax* infection was diagnosed by light microscopy unless stated otherwise. Results are presented for each antigen stratified by study design: cross-sectional and case–control studies, to identify markers of *P. vivax* infection, and cohort studies, to identify antibody responses that protect against *P. vivax* malaria.

### Association between antibody responses to *Pv*CSP repeat region and *P. vivax*

Circumsporozoite protein (CSP), the predominant surface protein of the pre-erythrocytic, sporozoite stage parasite, has been implicated in the invasion of hepatocytes [41]. *P. vivax* CSP contains a highly immunogenic central repeat domain flanked by amino and carboxyl sequences, which include highly conserved protein stretches (Regions I and II-plus). Three main allelic forms of *Pv*CSP, differing mainly in the central repeat region, have been described: VK210, VK247 [42], and *P. vivax*-like [43].

#### Cross-sectional and case–control studies

**Figure 2:**
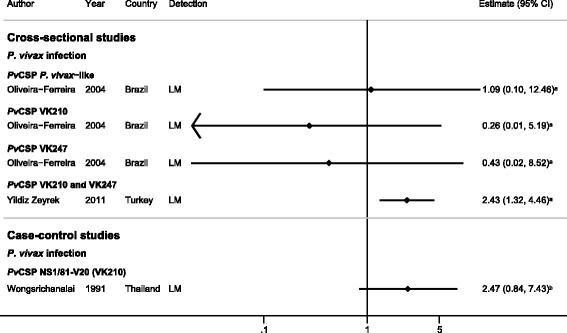
**Forest plot of the association of** ***Pv*** **CSP IgG responses with** ***Plasmodium vivax*** **infection.** Estimates represent the odds of *P. vivax* infection in IgG responders compared with non-responders. ^**a**^Data supplied by the original authors and estimate calculated by the current authors; ^**b**^published estimate. All estimates are unadjusted. Abbreviations: LM, light microscopy; W, weight.

Two cross-sectional studies [28,39] and one case–control study [38] examined the association of antibody responses to the *Pv*CSP repeat region and *P. vivax* infection. A cross-sectional study in Brazil showed no association between IgG responses to the *P. vivax*-like allele of *Pv*CSP and odds of *P. vivax* infection (OR = 1.09, responders versus non-responders), but IgG responders to VK210 or VK247 alleles had a non-significant reduction of 74% and 57% respectively, in the odds of *P. vivax* infection compared with non-responders (Figure 2) [28]. By contrast, a cross-sectional study in Turkey showed a 2.4-fold increase in the odds of *P. vivax* infection in IgG responders to combined VK210 and VK247 alleles (compared with non-responders, Figure 2) [39]. Similar associations were also seen for IgM (OR = 3.8, 95% CI 2.06 to 7.01) [39]. A case–control study in Thailand also showed a similar 2.4-fold increase in the odds of *P. vivax* infection in IgG responders to the NS1/81-V20 antigen, which includes the CSP repetitive domain (VK210 allele) compared with non-responders (Figure 2) [38].

### Association between antibody responses to *Pv*DBP and *Plasmodium vivax*

*Pv*DBP is a leading vaccine candidate because invasion of erythrocytes is largely dependent upon its interaction with the Duffy blood-group antigen [44]. The conserved N-terminal cysteine-rich rich region II (*Pv*DBPII) constitutes the receptor binding domain of *Pv*DBP [45,46]. The full-length ectodomain of *Pv*DBP, comprising regions II to VI, is thought to correspond to the soluble form of the protein [47].

### *Pv*DBP region II

#### Cross-sectional studies

**Figure 3:**
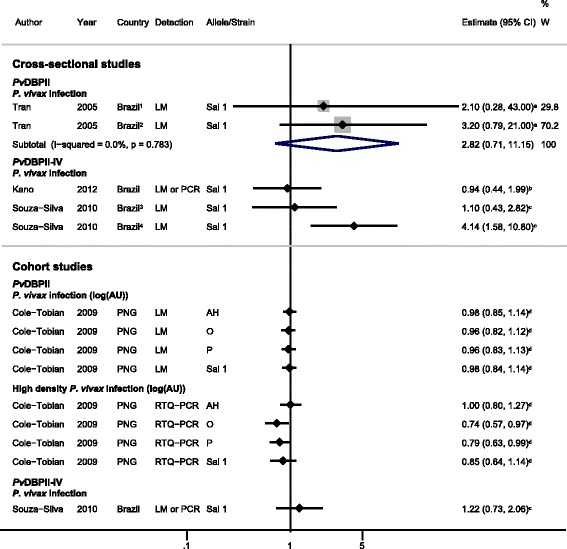
**Forest plot of the association of** ***Pv*** **DBP IgG responses with** ***Plasmodium vivax*** **infection.** Estimates represent the estimate of *P. vivax* infection in IgG responders compared with non-responders, unless stated otherwise. For cross-sectional studies, the estimate is an odds ratio; for cohort studies, it is a risk ratio. ^**1**^Colina study site; ^**2**^Ribeirinha study site; ^**3**^First (baseline) survey; ^**4**^Second survey. ^**a**^Estimate supplied by the original authors following correspondence; ^**b**^data supplied by the original authors and estimate calculated by the current authors; ^**c**^estimate calculated by the current authors from data in the paper; ^**d**^published estimate. All estimates are unadjusted, with the exception of estimates from Cole-Tobian *et al*. [34], which were adjusted for age. When *I* ^2^ was ≤30%, meta-analysis based on a fixed-effects model was conducted. Estimates for distinct alleles of *Pv*DBPII were not combined in meta-analysis. Abbreviations: AU, antigen units; LM, light microscopy; RTQ-PCR, real-time quantitative polymerase chain reaction; PNG, Papua New Guinea; W, weight.

The association of antibodies to *Pv*DBP region II and *P. vivax* infection was examined in one cross-sectional study and one cohort study [30,34]. The cross-sectional study was conducted at two study sites in Brazil, and pooled OR showed that IgG responders to *Pv*DBPII Sal 1 strain had higher odds of *P. vivax* infection compared with non-responders (pooled OR using fixed effects (feOR) = 2.82, 95% CI 0.71 to 11.15, *I*^2^ = 0%) (Figure 3) [30] indicating that this antigen may be indicative of exposure in this population.

#### Cohort studies

A cohort study conducted in PNG by Cole-Tobian *et al.* [34] revealed no evidence for an association between IgG responses to any of the *Pv*DBPII alleles studied (AH, O, P, or Sal 1) and protection against *P. vivax* infection detected by light microscopy (log_(antigen units + 1)_; HR ranged from 0.96 to 0.98, Figure 3) or LDR-FMA (log_(antigen units + 1)_; HR ranged from 0.92 to 1.02) [34]. However, IgG responders to *Pv*DBPII (O, P, and Sal 1 alleles) had a lower risk of high-density *P. vivax* infections (>150 parasites/μl) compared with non-responders (HR ranged from 0.74 to 0.85) (Figure 3) [34]. In the same Papua New Guinean cohort, King *et al.* [35] tested plasma samples for their ability to inhibit binding of *Pv*DBPII to its receptor, Duffy antigen: individuals with high-level (>90%) binding inhibitory activity had a 55% reduction in risk of *P. vivax* infection detected by light microscopy compared with those with low-level (<50%) binding inhibitory activity (HR = 0.45, 95% CI 0.2 to 0.98) [35], providing further evidence of *Pv*DBPII as a target of protective antibodies.

### *Pv*DBP region II-IV

#### Cross-sectional studies

The association of antibodies to *Pv*DBP region II-IV (*Pv*DBPII-IV) and *P. vivax* infection was examined in one cross-sectional study [23] and one cohort study that also provided two sets of cross-sectional data [29]. In a cross-sectional study, Kano *et al.* [23] found no evidence for any association between IgG responses to *Pv*DBPII-IV Sal 1 and *P. vivax* infection. One study by Souza-Silva *et al.* provided data from two cross-sectional surveys conducted in the same study site in Brazil [29]. While the first (baseline) survey provided no evidence for an association between IgG responses to *Pv*DBPII-IV Sal 1 and *P. vivax* infection detected by light microscopy (OR = 1.10, 95% CI 0.43 to 2.82), the second survey showed that the IgG responders had increased odds of *P. vivax* infection compared with non-responders (OR = 4.14, 95% CI 1.58 to 10.8) (Figure 3) [29]. Owing to significant heterogeneity, these estimates were not combined (*I*^2^ = 67.6%, *P* = 0.046).

#### Cohort studies

Cohort data from Souza-Silva *et al.* [29] found a 22% increased prospective risk of *P. vivax* infection (detected by light microscopy or PCR) in *Pv*DBPII-IV IgG responders compared with non-responders (RR = 1.22, 95% CI 0.73 to 2.06, Figure 3). These results suggest that *P. vivax* infection during follow-up induced an anti-*Pv*DBPII-IV response.

### Association between antibody responses to *Pv*MSP-1 and *P. vivax*

MSP-1 is conserved in all *Plasmodium* species, and is thought to be essential for blood-stage development of the parasite [48]. *Pv*MSP-1 has a polymorphic N-terminus (*Pv*MSP-1_NT_) and a relatively conserved C-terminus [49]. Studies in *P. falciparum* have established that post-translational proteolytic processing of *Pf*MSP-1 generates four fragments, including a C-terminal 42 kDa fragment, which is further processed into a 19 kDa fragment that remains on the surface of the merozoite during invasion (*Pv*MSP-1_19_) [50-52].

### *Pv*MSP-1_19_

A total of eight studies investigated responses to *Pv*MSP-1_19_ and *P. vivax* outcomes [22,23,26,27,32,34,39,40].

#### Cross-sectional studies

**Figure 4:**
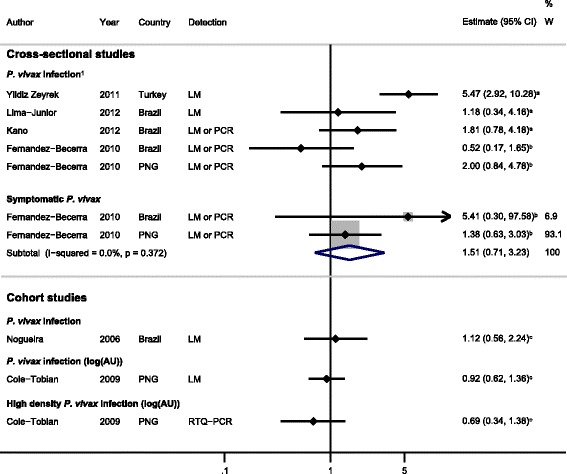
**Forest plot of the association of** ***Pv*** **MSP-1**_**19**_ **IgG responses with** ***Plasmodium vivax*** **outcomes.** Estimates represent the estimate of *P. vivax* infection in IgG responders compared with non-responders unless stated otherwise. For cross-sectional and case–control studies, the estimate is an odds ratio; for cohort studies, it is a risk ratio. ^**1**^Meta-analysis of IgG responses to *Pv*MSP-1_19_ and odds of *P. vivax* infection (estimates from cross-sectional studies) showed a high degree of heterogeneity (*I* ^2^ = 73.8%, *P* = 0.004), so results were not pooled. ^**a**^Data supplied by the original authors, and estimate calculated by the current authors; ^**b**^estimate calculated by the current authors from data in the paper; ^**c**^published estimate. All estimates are unadjusted, with the exception of estimates from Cole-Tobian *et al*. [34], which were adjusted for age, and Noguiera *et al*. [27], which were adjusted for geographical sector. When *I* ^2^ was ≤30%, meta-analysis based on a fixed-effects model was conducted. AU, antigen units; LM, light microscopy; PCR, polymerase chain reaction; PNG, Papua New Guinea; RTQ-PCR, real-time quantitative PCR; W, weight.

Four cross-sectional studies (providing five sets of data) investigated the association between IgG responders to *Pv*MSP-1_19_ and *P. vivax* infection (Figure 4) [22,23,26,39]. Meta-analysis revealed significant heterogeneity between studies (*I*^2^ = 73.8%, *P* = 0.004), so a pooled estimate was not reported. Increased odds of *P. vivax* infection (diagnosed by light microscopy or by LM in combination with PCR) in *Pv*MSP-1_19_ IgG responders compared with non-responders was found in Brazil (increased odds of 81% [23] and 18% [26]), Turkey (447% increased odds [39]) and Papua New Guinea (100% increased odds [22]) (Figure 4). Conversely, data from another Brazilian study [22] showed that IgG responders to *Pv*MSP-1_19_ had 48% decreased odds of *P. vivax* infection (compared with non-responders) [22]. However, when these authors investigated the outcome of symptomatic *P. vivax* malaria at sites in Brazil and PNG, they found that IgG responders to *Pv*MSP-1_19_ had 51% increased odds of symptomatic *P. vivax* (feOR =1.51, 95% CI 0.71 to 3.23, *I*^2^ = 0%; Figure 4) [22]. Overall, these data, taken together support IgG response against *Pv*MSP-1_19_ as a marker of *P. vivax* infection in geographically diverse populations (Figure 4).

Two studies looked at IgM responses, as a marker of recent exposure to *Pv*MSP-1_19_. Yildiz Zeyrek *et al.* [39] showed that IgM responses were associated with higher odds of *P. vivax* infection and of increased magnitude compared with IgG (responders compared with non-responders, OR = 48.8, 95% CI 16.3 to 146.1). One cross-sectional study in Indonesia, which examined combined IgG and IgM responses (and was therefore not included in the IgG or IgM meta-analyses), showed a 71% reduction in the odds of *P. vivax* infection in responders versus non-responders (OR = 0.29, 95% CI 0.09 to 0.88) [32].

#### Cohort studies

IgG against *Pv*MSP-1_19_ as a marker of protective immunity was assessed in two cohort studies [27,34]. No evidence for an association between *Pv*MSP-1_19_ IgG responses and *P. vivax* infection was found in Brazil (responders versus non-responders; RR = 1.12, 95% CI 0.56 to 2.24) [27] or Papua New Guinea (log_(antigen units + 1)_; light microscopy: RR = 0.92, 95% CI 0.62 to 1.36 (Figure 4); LDR-FMA: RR = 1.07, 95% CI 0.78 to 1.47) [34]. Although Cole-Tobian *et al.* [34] found no association with *P. vivax* infection in Papua New Guinea, they did observe a 31% reduced risk of high-density *P. vivax* infection (≥150 parasites/μl) (log_(antigen units + 1)_; RR = 0.69, 95% CI 0.34 to 1.38).

### *Pv*MSP-1 N-terminus

#### Cross-sectional studies

**Figure 5:**
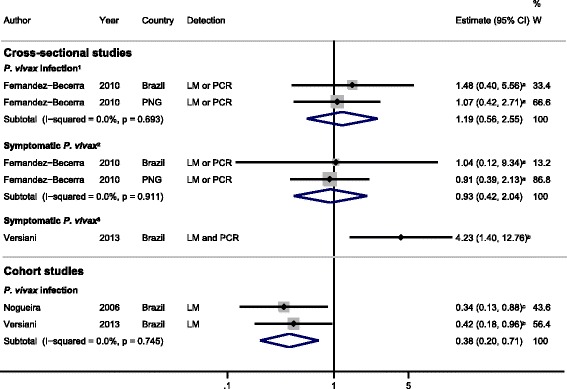
**Forest plot of the association of** ***Pv*** **MSP-1 N-terminus IgG responses with** ***Plasmodium vivax*** **outcomes.** Estimates represent the estimate of *P. vivax* infection in IgG responders compared with non-responders. For cross-sectional studies, the estimate is an odds ratio; for cohort studies, it is a risk ratio. ^**1**^Symptomatic and asymptomatic *P. vivax*-positive individuals were compared with *P. vivax*-negative individuals; ^**2**^symptomatic individuals who were positive for *P. vivax* were compared with asymptomatic individuals who were either positive or negative for *P. vivax*; ^**3**^symptomatic individuals who were positive for *P. vivax* by both PCR and LM were compared with individuals who were negative for *P. vivax* by both PCR and LM. ^**a**^Estimate calculated by the current authors from data in the paper; ^**b**^data supplied by the original authors and estimate calculated by the current authors; ^**c**^published estimate. All estimates are unadjusted, with the exception of the estimate from Nogueira *et al*. [27], which was adjusted for geographical sector. When *I* ^2^ was ≤30%, meta-analysis based on a fixed-effects model was conducted. Abbreviations: LM, light microscopy; PNG, Papua New Guinea; W, weight.

The cross-sectional study by Fernandez-Becerra *et al.* [22], conducted in Brazil and PNG, also investigated responses against the N-terminus of *Pv*MSP-1 (*Pv*MSP-1_NT_) and found no evidence for an association between IgG response (responders versus non-responders) to *Pv*MSP-1_NT_ and either *P. vivax* infection or symptomatic *P. vivax* (feOR = 1.19, 95% CI 0.56 to 2.55, *I*^2^ = 0% and feOR = 0.93, 95% CI 0.42 to 2.04, *I*^2^ = 0%, respectively; Figure 5). By contrast, cross-sectional data from Versiani *et al.* [31] in Brazil showed that IgG responders to *Pv*MSP-1_NT_ had 4.2-fold increased odds of developing symptomatic *P. vivax* (PCR- and light microscopy-positive) compared with asymptomatic *P. vivax* malaria (PCR-positive and light microscopy-negative) (OR = 4.23, 95% CI 1.40 to 12.76, Figure 5).

#### Cohort studies

Meta-analysis of two cohort studies in Brazil showed that IgG responders to *Pv*MSP-1_NT_ had a 62% reduced risk of *P. vivax* infection compared with non-responders (feOR = 0.38, 95% CI 0.20 to 0.71, *I*^2^ = 0%, Figure 5) [27,31] indicating that *Pv*MSP-1_NT_ may be a target for protective immunity.

### Association between antibody responses to *Pv*MSP-3α and *P. vivax*

Members of the *P. vivax* MSP-3 multigene family, including *Pv*MSP-3α, are structurally related to *P. falciparum* MSP-3, and are thought to associate with proteins anchored to the merozoite surface [53]. *Pv*MSP-3α is highly polymorphic, with polymorphisms clustered in the N-terminal half of the central alanine-rich coiled-coil domain (Block I repeats, *Pv*MSP-3α_RI_) and the less variable C-terminal half of the domain (Block II repeats, *Pv*MSP-3α_RII_). By contrast, the extreme N-terminal (*Pv*MSP-3α_NT_) and C-terminal (*Pv*MSP-3α_CT_) domains are relatively conserved [54].

#### Cross-sectional studies

**Figure 6:**
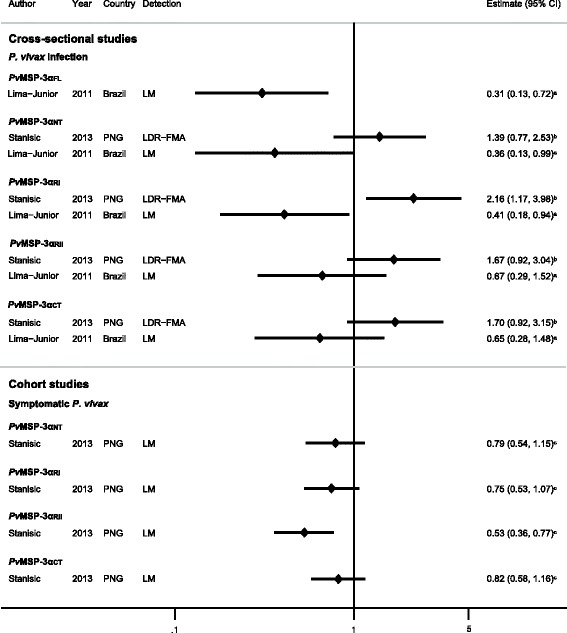
**Forest plot of the association of** ***Pv*** **MSP-3α IgG responses with** ***Plasmodium vivax*** **outcomes.** Estimates represent the estimate of *P. vivax* infection in IgG responders compared with non-responders. For cross-sectional studies, estimate is an odds ratio; for cohort studies, it is a risk ratio. ^**a**^Data supplied by the original authors and estimate calculated by the current authors; ^**b**^estimate calculated by the current authors from data in the paper; ^**c**^published estimate. All estimates are unadjusted, with the exception of estimates from cohort data from Stanisic *et al*. [36], which were adjusted for age, season, spatial variation, and individual differences in exposure. Meta-analysis of responses to *Pv*MSP-3α_NT_, *Pv*MSP-3α_RI_, *Pv*MSP-3α_RII_, and *Pv*MSP-3α_CT_, and odds of *P. vivax* infection (estimates from cross-sectional studies) showed a high degree of heterogeneity (*I* ^2^ > 75% and/or *P* < 0.1) so estimates were not pooled. Abbreviations: LM, light microscopy; LDR-FMA, ligase detection reaction-fluorescent microsphere assay; PNG, Papua New Guinea.

One cross-sectional study in Brazil by Lima-Junior *et al.*[25] and one cohort study in PNG by Stanisic *et al.* [36] (which contributed both cross-sectional and cohort data) investigated the association between antibody responses to regions of *Pv*MSP-3α and *P. vivax* outcomes. The two cross-sectional studies gave opposing results and were not combined (*I*^2^ > 75% and/or *P* < 0.01). Lima-Junior *et al.* [25] showed around a 60% reduction in the odds of *P. vivax* infection in IgG responders to *Pv*MSP-3α full-length (MSP-3α_FL_), *Pv*MSP-3α_NT_, *Pv*MSP-3α_RI_, and around a 35% reduction for *Pv*MSP-3α_RII_ and *Pv*MSP-3α_CT_ compared with non-responders (Figure 6). Conversely, Stanisic *et al.* [36] showed fold increases of between 1.39 and 2.16 in the odds of *P. vivax* infection in PNG in IgG responders to *Pv*MSP-3α_NT_, *Pv*MSP-3α_RI_, *Pv*MSP-3α_RII_, and *Pv*MSP-3α_CT_, compared with non-responders (Figure 6).

#### Cohort studies

Although *Pv*MSP-3α antibodies appeared to be a marker of *P. vivax* infection in cross-sectional data from Stanisic *et al.* [36], cohort data from the same study showed a 47% reduction in the risk of symptomatic *P. vivax* for *Pv*MSP-3α_RII_ IgG responders and around a 20% risk reduction for IgG responders to *Pv*MSP-3α_NT_, *Pv*MSP-3α_RI_ and *Pv*MSP-3α_CT_ (Figure 6).

### Association between antibody responses to *Pv*MSP-5 and *P. vivax*

#### Case–control studies

The highly polymorphic *Pv*MSP-5 contains potential signal and glycosylphosphatidyl inositol (GPI) anchor sequences and a single EGF-like domain near the carboxyl-terminus [55]. *Pv*MSP-5 has been localized to the apical end of merozoites [56]. One case–control study in Indonesia showed no association between IgG (OR = 0.81, 95% CI 0.44 to 1.47) or IgM (OR = 1.12, 95% CI 0.62 to 2.04) responses to *Pv*MSP-5 and odds of symptomatic *P. vivax* [33].

### Association between antibody responses to *Pv*MSP-9 and *P. vivax*

*Pv*MSP-9 is associated with the surface of the merozoite and contains a hydrophobic signal sequence, a highly conserved N-terminal domain with a cluster of four cysteines, and a C-terminal region containing two species-specific blocks of repeats, designated *Pv*MSP-9_RI_ and *Pv*MSP-9_RII_ [57,58]. Recombinant proteins may represent individual blocks or both blocks (*Pv*MSP-9_RIRII_).

### *Pv*MSP-9 N-terminus

#### Cross-sectional studies

**Figure 7:**
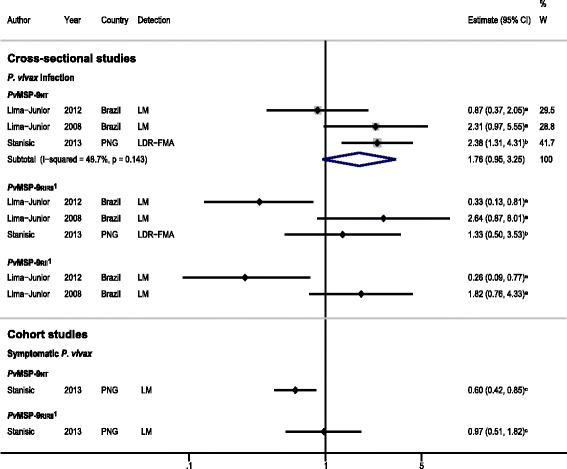
**Forest plot of the association of** ***Pv*** **MSP-9 IgG responses with** ***P. vivax*** **outcomes.** Estimates represent the estimate of *P. vivax* infection in IgG responders compared with non-responders. For cross-sectional studies, the estimate is an odds ratio, for cohort studies, it is a risk ratio. ^**a**^Data supplied by original authors and estimate calculated by the current authors; ^**b**^estimate calculated by the current authors from data in the paper; ^**c**^published estimate. All estimates are unadjusted, with the exception of estimates from cohort data from Stanisic *et al*. [36], which were adjusted for age, season, spatial variation, and individual differences in exposure. ^**1**^Meta-analysis of *Pv*MSP-9_RIIRII_ and *Pv*MSP-9_RII_ with odds of *P. vivax* infection showed a high degree of heterogeneity (*I* ^2^ = 77.5%, *P* = 0.012 and 87%, *P* = 0.006 respectively), so results were not pooled. Abbreviations: LDR-FMA, ligase detection reaction-fluorescent microsphere assay; LM, light microscopy; PNG, Papua New Guinea; W, weight.

Meta-analysis of three sets of cross-sectional data [24,26,36] showed that IgG responses to *Pv*MSP-9_NT_ were associated with a 76% increase in odds of *P. vivax* infection compared with non-responders (pooled OR using random effects reOR = 1.76, 95% CI 0.95 to 3.25, *I*^2^ = 48.7%, Figure 7) suggesting that *Pv*MSP-9_NT_ is a marker of exposure.

#### Cohort studies

One cohort study by Stanisic *et al.* [36] also provided evidence for a protective effect of IgG responses to *Pv*MSP-9_NT_, with a 40% reduction in the risk of symptomatic *P. vivax* (RR = 0.60, 95% CI 0.42 to 0.85).

### *Pv*MSP-9 block repeats

#### Cross-sectional studies

Three sets of cross-sectional data investigated the association between IgG antibodies to a protein representing the two blocks of repeats in *Pv*MSP-9 (*Pv*MSP-9_RIRII_) and *P. vivax* infection [24,26,36]. In a Brazilian study in 2012, Lima-Junior *et al.* [26] showed that IgG responders to *Pv*MSP-9_RIRII_ had a 67% reduction in the odds of *P. vivax* infection, compared with non-responders, but in a 2008 study by these authors [24] in the same region, *Pv*MSP-9_RIRII_ responders were found to have increased odds of *P. vivax* infection (OR = 2.64, Figure 7). Similar divergent results were also found in the 2008 study when IgG responses to *Pv*MSP-9_RII_ were examined [24]. Stanisic *et al.* [36] showed that IgG responders to *Pv*MSP-9_RIRII_ had a 33% increase in odds of *P. vivax* infection detected by LDR-FMA (OR = 1.33, 95% CI 0.50 to 3.53). Meta-analysis of *Pv*MSP-9_RIRII_ responses showed a high degree of heterogeneity in estimates (I^2^ = 77.5%, *P* = 0.012), and a pooled estimate was not reported.

#### Cohort studies

Cohort data from Stanisic *et al.* [36] showed no association between IgG responses to *Pv*MSP-9_RIRII_ and prospective risk of symptomatic *P. vivax* (RR = 0.97, 95% CI 0.51 to 1.82; Figure 7).

### Association between antibody responses to *Pv*AMA1 ectodomain and *P. vivax*

#### Cross-sectional and case–control studies

**Figure 8:**
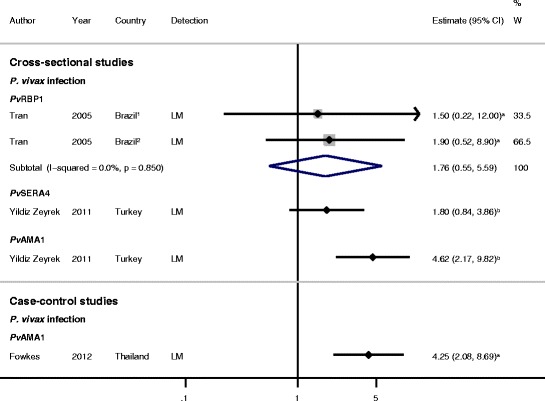
**Forest plot of the association of** ***Pv*** **AMA1,** ***Pv*** **RBP1, and** ***Pv*** **SERA4 IgG responses with** ***Plasmodium vivax*** **infection.** Estimates represent the odds of *P. vivax* infection in responders compared with non-responders. ^**1**^Colina study site; ^**2**^Ribeirinha study site. ^**a**^Estimate supplied by original authors following correspondence; ^**b**^data supplied by original authors and estimate calculated by the current authors. All estimates are unadjusted, with the exception of the estimate from Fowkes *et al*. [37] which was adjusted for gravidity, trimester, and prophylaxis, and the estimates from Tran *et al*. [30], which were adjusted for age. When *I* ^2^ was ≤30%, meta-analysis based on a fixed-effects model was conducted. Abbreviations: LM, light microscopy; W, weight.

*Pv*AMA1 is a type 1 transmembrane protein present in the microneme organelles of *Plasmodium* spp. A cross-sectional study in Turkey, which included both children and adults, investigated the association between anti-*Pv*AMA1 ectodomain responses and prevalence of *P. vivax* infection, and found that total IgG responses (OR = 4.62, 95% CI 2.17 to 9.82, Figure 8) and IgM responses (OR = 2.22, 95% CI 1.06 to 4.67) were associated with increased odds of *P. vivax* infection [39]. A similar increase in the odds of *P. vivax* infection was seen in a nested case–control study [37] of pregnant women in Thailand (responders versus non-responders, OR = 4.25, 95% CI 2.08 to 8.70, Figure 8). *Pv*AMA1 is therefore associated with *P. vivax* exposure in geographically and demographically diverse populations.

### Association between antibody responses to *Pv*RBP1 and *P. vivax*

#### Cross-sectional studies

*Pv*RBP1 is a Type I integral membrane protein, which was identified based on its ability to adhere preferentially to reticulocyte-enriched populations of erythrocytes [59,60]. Together with *Pv*RBP2, it is thought to form a complex at the apical pole of the merozoite [59,61]. Meta-analysis of two cross-sectional sets of data from two study sites by Tran *et al.* [30] indicated that IgG responders to *Pv*RBP1 had a 76% increase in odds of *P. vivax* infection detected by light microscopy compared with non-responders (feOR = 1.76, 95% CI 0.55 to 5.59, *I*^2^ = 0%, Figure 8).

### Association between antibody responses to *Pv*SERA4 and *P. vivax*

#### Cross-sectional studies

Serine repeat antigen 4 (SERA4) is the most dominantly expressed member of the *P. vivax SERA* multigene family, and its expression profile parallels that of *Pf*SERA5, a blood-stage vaccine candidate [62]. A single cross-sectional study investigating the association between anti-*Pv*SERA4 responses and *P. vivax* infection showed that both IgG and IgM responders had higher odds of *P. vivax* detected by light microscopy compared with non-responders (OR = 1.80, 95% CI 0.84 to 3.86, Figure 8 and OR = 2.50, 95% CI 1.39 to 4.49, respectively) [39].

## Discussion

In this systematic review, we aimed to identify immunological biomarkers of *P. vivax* infection and protective immunity by standardizing estimates of the association between *P. vivax* antibodies and *P. vivax* outcomes across populations. We found a paucity of studies investigating associations between antibody responses to *P. vivax* antigens and risk of *P. vivax*, particularly cohort studies, and studies conducted in the Asia-Pacific [1]. Although there was considerable heterogeneity between studies, antibody responses to several antigens were associated with *P. vivax* infection and protective immunity to *P. vivax*. However, this review highlights the need for additional studies, and identifies several issues in the interpretation and reporting of data from epidemiological studies investigating immunity to *P. vivax*.

Studies included in the review represented diverse geographical populations living in areas of varying *P. vivax* endemicity. However, the geographical regions and countries represented were limited. Half of the studies provided data from the Asia-Pacific region, which represents 91% of the population at risk of *P. vivax* malaria [1], but only four countries were represented (Indonesia, Turkey, Thailand, and Papua New Guinea). The remaining half of the studies provided data from South America, representing only 6% of the population at risk of *P. vivax* malaria [1], but all were performed in Brazil. This predominance of data from Brazil has implications for the generalizability of findings to other *P. vivax*-endemic regions in South America and the Asia-Pacific. However, despite the population heterogeneity and the considerable heterogeneity in estimates observed, immunological markers of *P. vivax* infection could be identified: IgG responses to *Pv*CSP, *Pv*MSP-1_19_, *Pv*MSP-9_NT_, and *Pv*AMA1 were associated with increased odds of *P. vivax* in geographically diverse populations. Other antigens were also shown to be markers of *P. vivax* infection, but only in single populations (*Pv*MSP-3α, *Pv*MSP-9_RIRII_, *Pv*DBP, and *Pv*RBP1). Serosurveillance using *Pv*CSP in Korea [63-67] and *Pv*MSP-1_19_ and *Pv*AMA1 in Vanuatu [68], Cambodia [69], and Somalia [70] has been employed to successfully map *P. vivax* transmission, and data from this review support their use in serosurveillance campaigns. However, this review highlights that further studies, conducted in diverse geographical settings and including additional antigens, are needed to ensure the generalizability of results across different populations with variable *P. vivax* transmission.

Protective immunity could only be examined in a handful of cohort studies, all of which showed evidence for protective blood-stage antibodies targeting *Pv*MSP-1_19_, *Pv*MSP-1_NT_, *Pv*MSP-3α and *Pv*MSP-9_NT_ antigens but only in single geographical locations. This was also the case for *Pv*DBP, a prime vaccine target (because of its essential role in invasion) [44] that is currently in Phase I trials [5]. *Pv*DBP was examined in only two cohort studies (which looked at different regions) and only Cole-Tobian *et al.* [34] showed evidence of allele-specific *Pv*DBPII protective immunity against high-density parasitemia. Interestingly, no cohort study examined the protective effect of antibody responses to either the pre-erythrocytic antigen *Pv*CSP or the gametocyte antigen *Pv*s25. Both of these have previously been assessed in Phase I trials [6,71-73], and *Pf*CSP comprises the current Phase III *P. falciparum* vaccine RTS,S, which has demonstrated around 50% efficacy in young children and around 30% efficacy in infants [15,16]. This review shows that very few antigens meet the pre-clinical criteria for prioritizing candidate antigens (targets of protective immunity in humans) for vaccine development, which is particularly pertinent given the difficulties in meeting other *in vitro* pre-clinical criteria (demonstrating essential/important function, abundance, limited genetic diversity, inhibition of parasite growth, protection in animal models of infection) [7] because of difficulties in maintaining *P. vivax* in culture. In order to prioritize antigens for *P. vivax* vaccine development, further studies including additional antigens and established, clinically relevant end-points (for example, allele-specific responses with allele-specific end-points) are needed to provide valuable evidence for the role of particular *P. vivax* antigens in protective immunity.

The considerable heterogeneity observed in the estimates of association, which meant that the magnitude and the direction of effect estimates from different studies varied considerably, was a major issue in the meta-analyses, such that study estimates could not be reliably combined in some instances. Methodological diversity between studies may have contributed to the heterogeneity: antibody responses were measured in different ways (alleles, antigen preparation); *P. vivax* infection was determined using detection methods of varying sensitivities (PCR is more sensitive than light microscopy); and statistical methodology varied. Furthermore, the estimates from the majority of studies were unadjusted for potential confounders, and within-study bias may also have contributed to the heterogeneity observed. Transmission micro-epidemiology within study sites may be an important confounder, biasing the direction of effect in either way: individuals living in areas with the highest *P. vivax* exposure will acquire both biomarkers of exposure and protective immunity, but will also be at increased risk of future *P. vivax* infections. Study design may also be an important source of heterogeneity. The majority of studies were cross-sectional or case–control studies in which antibody responses and *P. vivax* outcomes had been determined at a single time point, in those with or without *P. vivax* outcome. Although we used this study design to identify immunological markers of *P. vivax* exposure, using data from a single time point has the potential to also capture a degree of protective immunity in the population. Indeed, we observed these types of divergent associations for several antigens, including studies by the same authors using the same methodology both in different populations [22] and within the same population [24,26]. These findings highlight the limitations of using cross-sectional data, particularly when interpreting and comparing data across populations with varying degrees of *P. vivax* endemicity and immunity.

Differences in *P. vivax* transmission and exposure history will result in differential acquisition of immunity, which will influence associations between *P. vivax* antibody responses and clinical outcomes. To reduce bias in the systematic review, we excluded studies on transmigrants and studies in which the majority of the population resided in a malaria-endemic area for a short time. This bias was highlighted in two studies, which met the respective inclusion criteria, both by Lima-Junior *et al.* and performed in the same region of Brazil [24,26]. *Pv*MSP-9 IgG responders were found to have increased odds of *P. vivax* infection in 2008 [24], but decreased odds of infection in 2012 [26]. However, the population composition changed between the two studies: in 2008, 82% of participants were indigenous to the malaria-endemic area, compared with only 59% in 2012 (J. Ferreira, personal communication). This may explain, in part, the differences observed, because in both studies, time of residence in the malaria-endemic area was positively correlated with the anti-*Pv*MSP-9 response [24,26]. Differential effects according to transmission were also anecdotally observed: one study by Yidez-Zeyrek *et al.* in Turkey [39] showed greater magnitudes of effect with IgM than with IgG responses (*Pv*MSP-1_19_ and *Pv*SERA4), indicating that individuals living in this *P. vivax*-endemic area had limited exposure to *P. vivax*. Interestingly, the ability of IgG to serve as a marker of exposure in this study was more than twice that of estimates from areas of higher *P. vivax* transmission (in Brazil and PNG), highlighting the potential for transmission intensity to influence results. Future studies should be aware of the potential confounding introduced by variations in *P. vivax* exposure and transmission intensity, particularly those conducted in areas in which *P. vivax* epidemiology is complicated by the presence of migrant workers or transient communities, which is common in *P. vivax*-endemic areas in South America and South-East Asia.

This review aimed to be as comprehensive as possible, and to identify all data by which an association between *P. vivax* responses and *P. vivax* outcomes could be examined. By contacting authors directly, we were able to obtain data from a further 11 studies for which data was not originally published. Commonly, these studies were descriptive in nature, comparing antibody prevalence in *P. vivax* infected versus uninfected individuals, with no quantification of the magnitude of effect. Consequently, many included studies were not sufficiently powered to detect a statistically significant association between antibody responses and *P. vivax* outcomes. Publication bias may also be an issue in the *P. vivax* immunity literature, which could not be assessed in this review because of the small number of studies in each analysis.

In this review, we also included total IgG subclasses, as well as IgG and IgM, to infer potential functional mechanisms, with similar associations seen with subclasses as to total IgG (see Additional file 5). IgG1 and IgG3 were the predominant subclasses to *P. vivax* antigens, and may function by opsonic phagocytosis [74], or by fixing complement. We found only one study that utilized a functional assay: King *et al.* [35] showed that binding inhibitory antibodies to *Pv*DBPII were associated with protection from *P. vivax* infection. The lack of a continuous culturing system for *P. vivax* currently prohibits the use of most types of functional assays, but will clearly be important in future studies to determine the relative role of various immune mechanisms in protection against *P. vivax*.

## Conclusion and future directions

In the absence of an *in vitro* system, population-based immunoepidemiology studies are pivotal to identify *P. vivax* antigens associated with protective immunity and exposure. This systematic review revealed antibody responses to several antigens that were associated with *P. vivax* infection and protective immunity. However, observations were often made in a small number of (sometimes single) studies, and further research is needed to validate these findings. More research is needed not only on *P. vivax* blood-stage antigens, but on sporozoite and gametocyte antigens, which are important markers of *P. vivax* transmission. Cohort studies are preferable, because they can be used to examine both markers of exposure and protective immunity. Future studies should aim to represent diverse populations, and special consideration in design and interpretation of findings should be given to studies in populations that contain considerable migrant sub-populations. Importantly, future studies should appropriately and comprehensively report data, and we have previously published guidelines to facilitate correct reporting of malaria immunoepidemiology observational studies (Proposed guidelines of the reporting of Malaria Immuno-epidemiology Observational Studies (MIOS guidelines) [11]). Additional well-reported studies, encompassing a wider geographical area, will provide a solid evidence base for *P. vivax* antigens in the use of vaccines and serosurveillance tools.

## Additional files

Additional file 1
**Prisma 2009 Checklist.**
Additional file 2
**Full search strategy for PubMed database.**
Additional file 3
**Details of excluded studies.**
Additional file 4
**Details of recombinant antigens featured in systematic review.**
Additional file 5
**IgG Subclass analysis.**

## Abbreviations

AMA1: apical membrane antigen 1
CI: confidence interval
CC: case–control
CS: cross-sectional
CSP: circumsporozoite protein
CT: C-terminal
DBP: Duffy binding protein
ELISA: enzyme-linked immunosorbent assay
FL: full-length
HR: hazard ratio
IRR: incident rate ratio
LDR-FMA: ligase detection reaction-fluorescent microsphere assay
LM: light microscopy
MSP: merozoite surface protein
NT: N-terminal
OR: odds ratio
*Pf*: *P. falciparum*
PNG: Papua New Guinea
*Pv*: *P. vivax*
RI: Block I repeats
RII: Block II repeats
RR: risk ratio
RBP1: reticulocyte binding protein-1
RTQ-PCR: real-time quantitative polymerase chain reaction
SERA4: serine repeat antigen 4
