# Supplementary material for: Immunological markers of Plasmodium vivax exposure and immunity: a systematic review and meta-analysis
Source: BMC Med. 2014 Sep 9;12:150. doi: 10.1186/s12916-014-0150-1 (PMC4172944; doi:10.1186/s12916-014-0150-1)
Supplement: Additional file 2 — Full search strategy for PubMed database. [file 12916_2014_150_MOESM2_ESM.pdf]

Additional file 2: Full search strategy for Pubmed database

Database	Search string	Qualifiers / Filters	Results
**Pubmed**	malaria and vivax and plasmodium and (immunoglobulin OR IgG OR antibody OR immunity OR rhoptry OR microneme OR sporozoite OR CSP OR circumsporozoite OR trap OR thrombospondin OR merozoite OR MSP OR AMA OR DBP OR duffy binding protein OR EBA OR EBP OR erythrocyte binding* OR EMP OR erythrocyte membrane protein OR RBL OR reticulocyte binding like proteins OR RBP OR reticulocyte binding protein OR VSA OR variant surface antigen OR VIR OR gametocyte OR (transmission blocking (immunity OR protein OR antibody)) OR Pvs25 OR ookinete surface protein OR Pvs28 OR sexual stage surface protein OR Pvs230 OR transmission-blocking target antigen)	Publication date: to 30-Nov-2013<br>Article types: journal article<br>Species: human	1,360
