# Supplementary material for: Immunological markers of Plasmodium vivax exposure and immunity: a systematic review and meta-analysis
Source: BMC Med. 2014 Sep 9;12:150. doi: 10.1186/s12916-014-0150-1 (PMC4172944; doi:10.1186/s12916-014-0150-1)
Supplement: Additional file 3 — Details of excluded studies. [file 12916_2014_150_MOESM3_ESM.pdf]

Additional file 3: Details of excluded studies

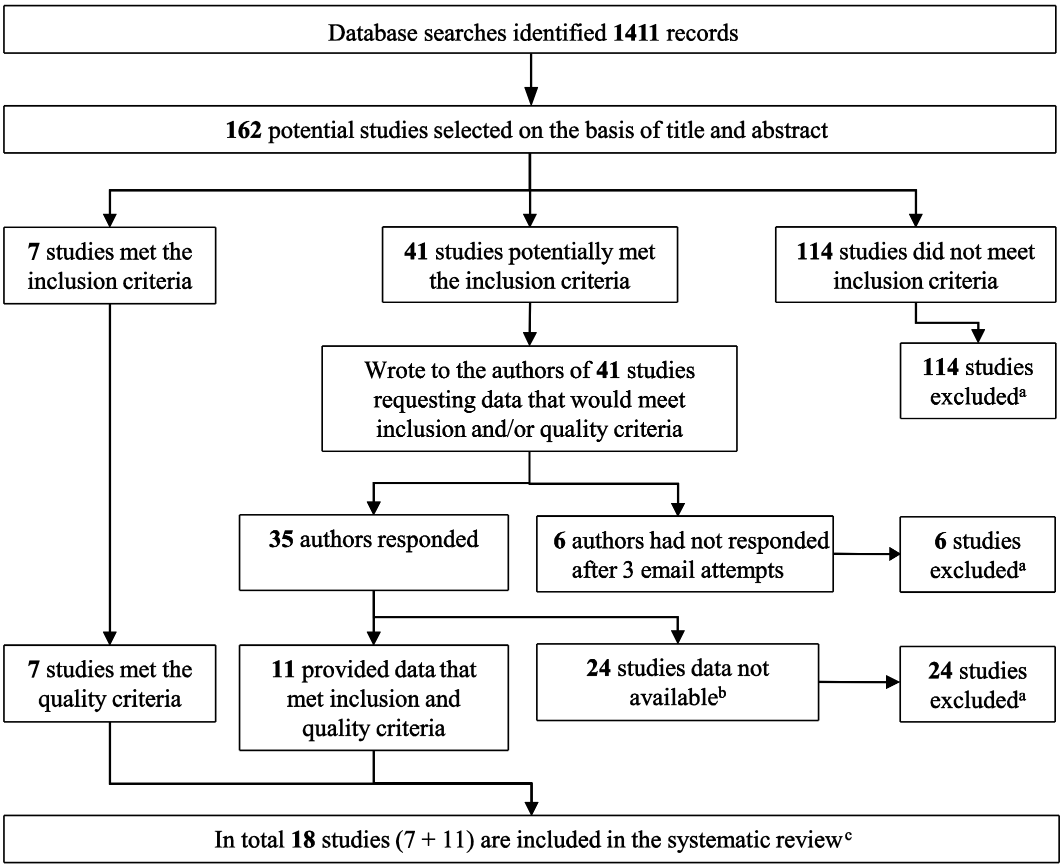

Figure 1 from manuscript

Inclusion and exclusion criteria for the systematic review are described on pages 7-9 of the manuscript. Briefly, population-based cohort, cross-sectional, case-control or treatment to reinfection studies that measured antibodies at enrolment were considered. Participants were required to have lived in *P. vivax*-endemic areas for a continuous period of 5 years.

After searching databases and examining the title and abstract, 162 potential studies were identified. Where there was insufficient information in the abstract to identify the characteristics of the study population, the paper was read in full to determine whether it met the inclusion criteria.

Seven out of 162 studies met the inclusion and quality criteria and were included in the systematic review [1-7].

Of the 162 studies examined, 114 records did not meet the inclusion criteria (**Figure 1**):

- 8 studies were excluded because all subjects were negative for *P. vivax* and there was therefore no outcome [8-15]
- 2 studies were excluded because the antibody data presented had been captured in a previous publication by the same authors [16, 17]
- 55 studies were excluded because antibody responses were either not reported or were determined for non-specific antigens such as whole parasites[18-72]
- 5 studies were excluded because antibody levels were measured in mosquitoes, not humans [73-77]
- 7 studies were excluded because more than 20% of the study population were migrants who had been living in the area for less than five years [78-84]
- 8 studies were excluded because they were conducted in non-endemic areas [85-92]
- 2 studies was excluded because antibody responses were tested using serum pooled from more than one individual [93, 94]
- 15 studies were excluded because recruitment of individuals was based on their clinical status (all *P. vivax* positive), and there was therefore no outcome as defined by this review [95-109]
- 3 studies were excluded because they were review articles and did not present new data [110-112]
- 1 study was excluded because it presented data from experimental *P. vivax* infections rather than from natural infections in an endemic area [113]

- 8 studies were excluded because of insufficient information regarding the origin of study samples or because controls were not representative of the population from which cases were drawn [114-121]

After reading the full text articles, 41 studies were considered to lack particular details or data to enable an assessment based on the inclusion and quality criteria [122-162]. The corresponding authors of these 41 studies were contacted to request additional study details or data to meet the inclusion and quality criteria of the review [122-162]:

- 6 studies were excluded because authors did not respond after three email attempts [131, 137, 139, 148, 153, 156]
- 24 studies were excluded after authors responded via email explaining that the requested data was not available [122-130, 133-135, 138, 141-143, 145, 146, 155, 158-162]
- 11 studies met the inclusion and quality criteria for the review after data was provided by the corresponding authors [132, 136, 140, 144, 147, 149-152, 154, 157]

References:

1. Cole-Tobian JL, Michon P, Biasor M, Richards JS, Beeson JG, Mueller I, King CL: **Strain-specific duffy binding protein antibodies correlate with protection against infection with homologous compared to heterologous plasmodium vivax strains in Papua New Guinean children**. *Infect and Immun* 2009, **77**(9):4009-4017.
2. Fernandez-Becerra C, Sanz S, Brucet M, Stanisic DI, Alves FP, Camargo EP, Alonso PL, Mueller I, del Portillo HA: **Naturally-acquired humoral immune responses against the N- and C-termini of the Plasmodium vivax MSP1 protein in endemic regions of Brazil and Papua New Guinea using a multiplex assay**. *Malar J* 2010, **9**:29.
3. Souza-Silva FA, Da Silva-Nunes M, Sanchez BAM, Ceravolo IP, Malafronte RS, Brito CFA, Ferreira MU, Carvalho LH: **Naturally acquired antibodies to Plasmodium vivax Duffy binding protein (DBP) in rural Brazilian Amazon**. *Am J Trop Med Hyg* 2010, **82**(2):185-193.
4. Woodberry T, Minigo G, Piera KA, Hanley JC, de Silva HD, Salwati E, Kenangalem E, Tjitra E, Coppel RL, Price RN, Anstey NM, Plebanski M: **Antibodies to**

Plasmodium falciparum and Plasmodium vivax merozoite surface protein 5 in Indonesia: species-specific and cross-reactive responses. *J Infect Dis* 2008, **198**(1):134-142.

5. Ak M, Jones TR, Charoenvit Y, Kumar S, Kaslow DC, Maris D, Marwoto H, Masbar S, Hoffman SL: **Humoral immune responses against Plasmodium vivax MSP1 in humans living in a malaria endemic area in flores, Indonesia**. *Southeast Asian J Trop Med Public Health* 1998, **29**(4):685-691.
6. Stanisic DI, Javati S, Kiniboro B, Lin E, Jiang J, Singh B, Meyer EV, Siba P, Koepfli C, Felger I, Galinski MR, Mueller, I: **Naturally Acquired Immune Responses to P. vivax Merozoite Surface Protein 3alpha and Merozoite Surface Protein 9 Are Associated with Reduced Risk of P. vivax Malaria in Young Papua New Guinean Children**. *PLoS Negl Trop Dis* 2013, **7**(11):e2498.
7. King CL, Michon P, Shakri AR, Marcotty A, Stanisic D, Zimmerman PA, Cole-Tobian JL, Mueller I, Chitnis CE: **Naturally acquired Duffy-binding protein-specific binding inhibitory antibodies confer protection from blood-stage Plasmodium vivax infection**. *Proc Natl Acad Sci U S A* 2008, **105**(24):8363-8368.
8. Turki H, Zoghi S, Mehrizi AA, Zakeri S, Raeisi A, Khazan H, Haghdoost AA: **Absence of Asymptomatic Malaria Infection in Endemic Area of Bashagard District, Hormozgan Province, Iran**. *Iran J Parasitol* 2012, **7**(1):36-44.
9. Pandey JP, Morais CG, Fontes CJ, Braga EM: **Immunoglobulin GM 3 23 5,13,14 phenotype is strongly associated with IgG1 antibody responses to Plasmodium vivax vaccine candidate antigens PvMSP1-19 and PvAMA-1**. *Malar J* 2010, **9**:229.
10. Maestre A, Muskus C, Duque V, Agudelo O, Liu P, Takagi A, Ntumngia FB, Adams JH, Sim KL, Hoffman SL, Corradin G, Velez ID, Wang R: **Acquired antibody responses against Plasmodium vivax infection vary with host genotype for duffy antigen receptor for chemokines (DARC)**. *PloS One* 2010, **5**(7):e11437.
11. Ceravolo IP, Bruna-Romero O, Braga EM, Fontes CJ, Brito CF, Souza JM, Krettli AU, Adams JH, Carvalho LH: **Anti-Plasmodium vivax duffy binding protein antibodies measure exposure to malaria in the Brazilian Amazon**. *Am J Trop Med Hyg* 2005, **72**(6):675-681.
12. Morais CG, Soares IS, Carvalho LH, Fontes CJ, Krettli AU, Braga EM: **IgG isotype to C-terminal 19 kDa of Plasmodium vivax merozoite surface protein 1 among subjects with different levels of exposure to malaria in Brazil**. *Parasitol Res* 2005, **95**(6):420-426.
13. Morais CG, Soares IS, Carvalho LH, Fontes CJ, Krettli AU, Braga EM: **Antibodies to Plasmodium vivax apical membrane antigen 1: persistence and correlation with malaria transmission intensity**. *Am J Trop Med Hyg* 2006, **75**(4):582-587.
14. Lee KN, Suh IB, Chang EA, Kim SD, Cho NS, Park PW, An SS, Park O, Lim C: **Prevalence of antibodies to the circumsporozite protein of Plasmodium vivax in five different regions of Korea**. *Trop Med Int Health* 2003, **8**(12):1062-1067.
15. Wang R, Arevalo-Herrera M, Gardner MJ, Bonelo A, Carlton JM, Gomez A, Vera O, Soto L, Vergara J, Bidwell SL, Domingo A, Fraser CM, Herrera S: **Immune responses to Plasmodium vivax pre-erythrocytic stage antigens in naturally exposed Duffy-negative humans: A potential model for identification of liver-stage antigens**. *Eur J Immunol* 2005, **35**(6):1859-1868.
16. Ceravolo IP, Souza-Silva FA, Fontes CJ, Braga EM, Madureira AP, Krettli AU, Souza JM, Brito CF, Adams JH, Carvalho LH: **Inhibitory properties of the antibody response to Plasmodium vivax Duffy binding protein in an area with unstable malaria transmission**. *Scand J Immunol* 2008, **67**(3):270-278.

17. Franke ED, Lucas CM, San Roman E, Wirtz RA: **Prevalence of antibody to the variant repeat of the circumsporozoite protein of Plasmodium vivax in Peru**. *The Am J Trop Med Hyg* 1992, **46**(6):708-710.
18. Cole-Tobian JL, Michon P, Dabod E, Mueller I, King CL: **Dynamics of asymptomatic Plasmodium vivax infections and Duffy binding protein polymorphisms in relation to parasitemia levels in Papua New Guinean children**. *Am J Trop Med Hyg* 2007, **77**(5):955-962.
19. Cole-Tobian JL, Biasor M, King CL: **High Complexity of *Plasmodium vivax* Infections in Papua New Guinean Children**. *Am J Trop Med Hyg* 2005, **73**(3):626-633.
20. da Silva-Nunes M, Ferreira MU: **Clinical spectrum of uncomplicated malaria in semi-immune Amazonians: beyond the "symptomatic" vs "asymptomatic" dichotomy**. *Mem Inst Oswaldo Cruz* 2007, **102**(3):341-347.
21. Danis-Lozano R, Rodriguez MH, Betanzos-Reyes AF, Hernandez-Avila JE, Gonzalez-Ceron L, Mendez-Galvan JF, Velazquez-Monroy OJ, Tapia-Conyer R: **Individual risk factors for Plasmodium vivax infection in the residual malaria transmission focus of Oaxaca, Mexico**. *Salud Publica Mex* 2007, **49**(3):199-209.
22. Das MK, Joshi H, Verma A, Singh SS, Adak T: **Malaria among the Jarawas, a primitive and isolated tribe on the Andaman islands, India**. *Ann Trop Med Parasitol* 2005, **99**(6):545-552.
23. de Araujo FCF, de Rezende AM, Fontes CJF, Carvalho LH, de Brito CFA: **Multiple-Clone Activation of Hypnozoites Is the Leading Cause of Relapse in Plasmodium vivax Infection**. *PloS One* 2012, **7**(11).
24. Ferreira MU, Karunaweera ND, da Silva-Nunes M, da Silva NS, Wirth DF, Hartl DL: **Population structure and transmission dynamics of plasmodium vivax in rural amazonia**. *J Infect Dis* 2007, **195**(8):1218-1226.
25. Figtree M, Pasay CJ, Slade R, Cheng Q, Cloonan N, Walker J, Saul A: **Plasmodium vivax synonymous substitution frequencies, evolution and population structure deduced from diversity in AMA 1 and MSP 1 genes**. *Mol Biochem Parasitol* 2000, **108**(1):53-66.
26. Gamage-Mendis AC, Rajakaruna J, Carter R, Mendis KN: **Transmission blocking immunity to human Plasmodium vivax malaria in an endemic population in Kataragama, Sri Lanka**. *Parasite Immunol* 1992, **14**(4):385-396.
27. Garzon-Ospina D, Lopez C, Forero-Rodriguez J, Patarroyo MA: **Genetic Diversity and Selection in Three Plasmodium vivax Merozoite Surface Protein 7 (Pvmsp-7) Genes in a Colombian Population**. *PloS One* 2012, **7**(9).
28. Genton B, D'Acremont Vr, Rare L, Baea K, Reeder JC, Alpers MP, Mueller I: **Plasmodium vivax and mixed infections are associated with severe malaria in children: a prospective cohort study from Papua New Guinea**. *PLoS Med* 2008, **5**(6):e127.
29. Gholizadeh S, Djadid ND, Basseri HR, Zakeri S, Ladoni H: **Analysis of von Willebrand factor A domain-related protein (WARP) polymorphism in temperate and tropical Plasmodium vivax field isolates**. *Malar J* 2009, **8**:137.
30. Gonzalez-Ceron L, Alvarado-Delgado A, Martinez-Barnetche J, Rodriguez MH, Ovilla-Munoz M, Perez F, Hernandez-Avila JE, Sandoval MA, Rodriguez Mdel C, Villarreal-Trevino C: **Sequence variation of ookinete surface proteins Pvs25 and Pvs28 of Plasmodium vivax isolates from Southern Mexico and their association to local anophelines infectivity**. *Infect Genet Evol* 2010, **10**(5):645-654.

31. Gopinath R, Wongsrichanalai C, Cordon-Rosales C, Mirabelli L, Kyle D, Kain KC: **Failure to detect a Plasmodium vivax-like malaria parasite in globally collected blood samples**. *J Infect Dis* 1994, **170**(6):1630-1633.
32. Gosi P, Khusmith S, Khalambaheti T, Lanar DE, Schaecher KE, Fukuda MM, Miller SR: **Polymorphism patterns in Duffy-binding protein among Thai Plasmodium vivax isolates**. *Malar J* 2008, **7**.
33. Grynberg P, Fernandes Fontes CJ, Braga EM: **Association between particular polymorphic residues on apical membrane antigen 1 (AMA-1) and platelet levels in patients with vivax malaria**. *Clin Microbiol Infect* 2007, **13**(11):1089-1094.
34. Gupta P, Das A, Singh OP, Ghosh SK, Singh V: **Assessing the genetic diversity of the vir genes in Indian Plasmodium vivax population**. *Acta Trop* 2012, **124**(2):133-139.
35. Han ET, Song TE, Park JH, Shin EH, Guk SM, Kim TY, Chai JY: **Allelic dimorphism in the merozoite surface protein-3alpha in Korean isolates of Plasmodium vivax**. *Am J Trop Med Hyg* 2004, **71**(6):745-749.
36. Han ET, Park JH, Shin EH, Choi MH, Oh MD, Chai JY: **Apical membrane antigen-1 (AMA-1) gene sequences of re-emerging Plasmodium vivax in South Korea**. *Korean J Parasitol* 2002, **40**(3):157-162.
37. Han GD, Zhang XJ, Zhang HH, Chen XX, Huang BC: **Use of PCR/DNA probes to identify circumsporozoite genotype of Plasmodium vivax in China**. *Southeast Asian J Trop Med Public Health* 1999, **30**(1):20-23.
38. Han ET, Lee WJ, Sattabongkot J, Jang JW, Nam MH, An SS, Suh I, Lim CS: **Sequence polymorphisms of Plasmodium vivax ookinete surface proteins (Pvs25 and Pvs28) from clinical isolates in Korea**. *Trop Med Int Health* 2010, **15**(9):1072-1076.
39. Henry-Halldin CN, Sepe D, Susapu M, McNamara DT, Bockarie M, King CL, Zimmerman PA: **High-throughput molecular diagnosis of circumsporozoite variants VK210 and VK247 detects complex Plasmodium vivax infections in malaria endemic populations in Papua New Guinea**. *Infect Genet Evol* 2011, **11**(2):391-398.
40. Ju HL, Kang JM, Moon SU, Bahk YY, Cho PY, Sohn WM, Park YK, Park JW, Kim TS, Na BK: **Genetic diversity and natural selection of Duffy binding protein of Plasmodium vivax Korean isolates**. *Acta Trop* 2013, **125**(1):67-74.
41. Ju HL, Kang JM, Moon SU, Kim JY, Lee HW, Lin K, Sohn WM, Lee JS, Kim TS, Na BK: **Genetic polymorphism and natural selection of Duffy binding protein of Plasmodium vivax Myanmar isolates**. *Malar J* 2012, **11**:60.
42. Khusmith S, Tapchaisri P, Tharavanij S, Bunnag D: **Antigenic diversity of Plasmodium vivax and their geographic distribution in Thailand**. *Southeast Asian J Trop Med Public Health* 1998, **29**(3):512-518.
43. Koepfli C, Ross A, Kiniboro B, Smith TA, Zimmerman PA, Siba P, Mueller I, Felger I: **Multiplicity and diversity of Plasmodium vivax infections in a highly endemic region in Papua New Guinea**. *PLoS Negl Trop Dis* 2011, **5**(12):e1424.
44. Leclerc MC, Gauthier C, Villegas L, Urdaneta L: **Genetic diversity of merozoite surface protein-1 gene of Plasmodium vivax isolates in mining villages of Venezuela (Bolivar State)**. *Acta Trop* 2005, **95**(1):26-32.
45. Lin E, Kiniboro B, Gray L, Dobbie S, Robinson L, Laumaea A, Schopflin S, Stanisic D, Betuela I, Blood-Zikursh, Siba P, Felger I, Schofield L, Zimmerman P, Mueller I: **Differential Patterns of Infection and Disease with P. falciparum and P. vivax in Young Papua New Guinean Children**. *PloS One* 2010, **5**(2):e9047.

46. McKenzie FE, Wongsrichanalai C, Magill AJ, Forney JR, Permpanich B, Lucas C, Erhart LM, O'Meara WP, Smith DL, Sirichaisinthop J, Gasser RA Jr: **Gametocytemia in Plasmodium vivax and Plasmodium falciparum infections**. *J Parasitol* 2006, **92**(6):1280-1285.
47. Mendis KN, Munesinghe YD, de Silva YN, Keragalla I, Carter R: **Malaria transmission-blocking immunity induced by natural infections of Plasmodium vivax in humans**. *Infect Immun* 1987, **55**(2):369-372.
48. Mendis C, Gamage-Mendis AC, De Zoysa AP, Abhayawardena TA, Carter R, Herath PR, Mendis KN: **Characteristics of malaria transmission in Kataragama, Sri Lanka: a focus for immuno-epidemiological studies**. *Am J Trop Med Hyg* 1990, **42**(4):298-308.
49. Moon SU, Lee HW, Kim JY, Na BK, Cho SH, Lin K, Sohn WM, Kim TS: **High frequency of genetic diversity of Plasmodium vivax field isolates in Myanmar**. *Acta Trop* 2009, **109**(1):30-36.
50. Nardin EH, Nussenzweig V, Nussenzweig RS, Collins WE, Harinasuta KT, Tapchaisri P, Chomcharn Y: **Circumsporozoite proteins of human malaria parasites Plasmodium falciparum and Plasmodium vivax**. *J Exp Med* 1982, **156**(1):20-30.
51. Ntumngia FB, Schloegel J, Barnes SJ, McHenry AM, Singh S, King CL, Adams JH: **Conserved and variant epitopes of Plasmodium vivax Duffy binding protein as targets of inhibitory monoclonal antibodies**. *Infect Immun* 2012, **80**(3):1203-1208.
52. Orjuela-Sánchez P, da Silva NS, da Silva-Nunes Mn, Ferreira MU: **Recurrent Parasitemias and Population Dynamics of Plasmodium vivax Polymorphisms in Rural Amazonia**. *Am J Trop Med Hyg* 2009, **81**(6):961-968.
53. Ramsey JM, Salinas E, Rodriguez MH: **Acquired transmission-blocking immunity to Plasmodium vivax in a population of southern coastal Mexico**. *Am J Trop Med Hyg* 1996, **54**(5):458-463.
54. Ranawaka MB, Munesinghe YD, de Silva DM, Carter R, Mendis KN: **Boosting of transmission-blocking immunity during natural Plasmodium vivax infections in humans depends upon frequent reinfection**. *Infect Immun* 1988, **56**(7):1820-1824.
55. Sanchez MR, Ramirez JA, Rodriguez MH, O'Reilly F, Larralde C, Ortiz-Ortiz L: **Antibody response to Plasmodium vivax antigens in human malaria**. *Am J Trop Med Hyg* 1994, **50**(3):329-338.
56. Salwati E, Minigo G, Woodberry T, Piera KA, de Silva HD, Kenangalem E, Tjitra E, Coppel RL, Price RN, Anstey NM, Plebanski M: **Differential cellular recognition of antigens during acute Plasmodium falciparum and Plasmodium vivax malaria**. *J Infect Dis* 2011, **203**(8):1192-1199.
57. Sulzer AJ, Cantella R, Colichon A, Gleason NN, Walls KW: **A focus of hyperendemic Plasmodium malariae-P. vivax with no P. falciparum in a primitive population in the Peruvian Amazon jungle**. *Bull World Health Organ* 1975, **52**(3):273-278.
58. Tapchaisri P, Chomcharn Y, Poonthong C, Asavanich A, Limsuwan S, Maleevan O, Tharavanij S, Harinasuta T: **Anti-sporozoite antibodies induced by natural infection**. *Am J Trop Med Hyg* 1983, **32**(6):1203-1208.
59. Wickramarachchi T, Premaratne PH, Dias S, Handunnetti SM, Udagama-Randeniya PV: **Genetic complexity of Plasmodium vivax infections in Sri Lanka, as reflected at the merozoite-surface-protein-3 alpha locus**. *Ann Trop Med Parasitol* 2010, **104**(2):95-108.

60. Kain KC, Brown AE, Lanar DE, Ballou WR, Webster HK: **Response of Plasmodium vivax variants to chloroquine as determined by microscopy and quantitative polymerase chain reaction**. *Am J Trop Med Hyg* 1993, **49**(4):478-484.
61. Bonilla JA, Validum L, Cummings R, Palmer CJ: **Genetic diversity of Plasmodium vivax Pvcsp and Pvmsp1 in Guyana, South America**. *Am J Trop Med Hyg* 2006, **75**(5):830-835.
62. van der Kaay HJ, Klein F, Hagenaar-de Weerdt M, Meuwissen JH: **Immuno-epidemiology of malaria: a study in a tribal area of West Irian**. *Bull World Health Organ* 1973, **49**(3):267-274.
63. Tjitra E, Anstey NM, Sugiarto P, Warikar N, Kenangalem E, Karyana M, Lampah DA, Price RN: **Multidrug-resistant Plasmodium vivax associated with severe and fatal malaria: a prospective study in Papua, Indonesia**. *PLoS Med* 2008, **5**(6):e128.
64. de Arruda M, Nardin EH, Nussenzweig RS, Cochrane AH: **Sero-epidemiological studies of malaria in Indian tribes and monkeys of the Amazon Basin of Brazil**. *Am J Trop Med Hyg* 1989, **41**(4):379-385.
65. Nardin E, Nussenzweig R, McGregor I, Bryan J: **Antibodies to sporozoites: their frequent occurrence in individuals living in an area of hyperendemic malaria**. *Science* 1979, **206**(4418):597-599.
66. Arévalo-Herrera M, Solarte Y, Rocha L, Alvarez D, Beier JC, Herrera S: **Characterization of Plasmodium vivax transmission-blocking activity in low to moderate malaria transmission settings of the Colombian Pacific coast**. *Am J Trop Med Hyg* 2011, **84**(2 Suppl):71-77.
67. Danis-Lozano R, Rodriguez MH, Gonzalez-Ceron L, Hernandez-Avila M: **Risk factors for Plasmodium vivax infection in the Lacandon forest, southern Mexico**. *Epidemiol Infect* 1999, **122**(3):461-469.
68. de Arruda ME, Aragaki C, Gagliardi F, Haile RW: **A seroprevalence and descriptive epidemiological study of malaria among Indian tribes of the Amazon basin of Brazil**. *Ann Trop Med Parasitol* 1996, **90**(2):135-143.
69. Gonzalez JM, Hurtado S, Arevalo-Herrera M, Herrera S: **Variants of the Plasmodium vivax circumsporozoite protein (VK210 and VK247) in Colombian isolates**. *Mem Inst Oswaldo Cruz* 2001, **96**(5):709-712.
70. Khusmith S, Tharavanij S, Bunnag D: **Antigenic disparity of Plasmodium vivax causing initial symptoms and causing relapse**. *Southeast Asian J Trop Med Public Health* 1998, **29**(3):519-524.
71. Suh IB, Choi HK, Lee SW, Woo SK, Kang HY, Won YD, Cho M, Lim CS: **Reactivity of sera from cases of Plasmodium vivax malaria towards three recombinant antigens based on the surface proteins of the parasite**. *Ann Trop Med Parasitol* 2003, **97**(5):481-487.
72. Nichols ME, Rubinstein P, Barnwell J, Rodriguez de Cordoba S, Rosenfield RE: **A new human Duffy blood group specificity defined by a murine monoclonal antibody. Immunogenetics and association with susceptibility to Plasmodium vivax**. *J Exp Med* 1987, **166**(3):776-785.
73. Arevalo-Herrera M, Solarte Y, Zamora F, Mendez F, Yasnot MF, Rocha L, Long C, Miller LH, Herrera S: **Plasmodium vivax: transmission-blocking immunity in a malaria-endemic area of Colombia**. *Am J Trop Med Hyg* 2005, **73**(5 Suppl):38-43.
74. De Arruda ME, Collins KM, Hochberg LP, Ryan PR, Wirtz RA, Ryan JR: **Quantitative determination of sporozoites and circumsporozoite antigen in mosquitoes infected with Plasmodium falciparum or P-vivax**. *Ann Trop Med Parasitol* 2004, **98**(2):121-127.

75. Galardo AK, Arruda M, D'Almeida Couto AA, Wirtz R, Lounibos LP, Zimmerman RH: **Malaria vector incrimination in three rural riverine villages in the Brazilian Amazon**. *Am J Trop Med Hyg* 2007, **76**(3):461-469.
76. Hii JL, Smith T, Vounatsou P, Alexander N, Mai A, Ibam E, Alpers MP: **Area effects of bednet use in a malaria-endemic area in Papua New Guinea**. *Trans R Soc Trop Med Hyg* 2001, **95**(1):7-13.
77. Mehrizi AA, Zakeri S, Rafati S, Salmanian AH, Djadid ND: **Immune responses elicited by co-immunization of Plasmodium vivax and P. falciparum MSP-1 using prime-boost immunization strategies**. *Parasite Immunol* 2011, **33**(11):594-608.
78. Brown AE, Webster HK, Krinchai K, Gordon DM, Wirtz RA, Permpanich B: **Characteristics of natural antibody responses to the circumsporozoite protein of Plasmodium vivax**. *Am J Trop Med Hyg* 1991, **44**(1):21-27.
79. de Arruda M, Carvalho MB, Nussenzweig RS, Maracic M, Ferreira AW, Cochrane AH: **Potential vectors of malaria and their different susceptibility to Plasmodium falciparum and Plasmodium vivax in northern Brazil identified by immunoassay**. *Am J Trop Med Hyg* 1986, **35**(5):873-881.
80. Franke ED, Lucas CM, Chauca G, Wirtz RA, Hinostroza S: **Antibody response to the circumsporozoite protein of Plasmodium vivax in naturally infected humans**. *Am J Trop Med Hyg* 1992, **46**(3):320-326.
81. Maheswary NP, Perpanich B, Rosenberg R: **Presence of antibody to a heterologous circumsporozoite protein of Plasmodium vivax (VK247) in southeastern Bangladesh**. *Trans R Soc Trop Med Hyg* 1992, **86**(1):28.
82. Mertens F, Levitus G, Camargo LM, Ferreira MU, Dutra AP, Del Portillo HA: **Longitudinal study of naturally acquired humoral immune responses against the merozoite surface protein 1 of Plasmodium vivax in patients from Rondonia, Brazil**. *Am J Trop Med Hyg* 1993, **49**(3):383-392.
83. Park JW, Yoo SB, Oh JH, Yeom JS, Lee YH, Bahk YY, Kim YS, Lim KJ: **Diagnosis of vivax malaria using an IgM capture ELISA is a sensitive method, even for low levels of parasitemia**. *Parasitol Res* 2008, **103**(3):625-631.
84. Soares IS, Levitus G, Souza JM, Del Portillo HA, Rodrigues MM: **Acquired immune responses to the N- and C-terminal regions of Plasmodium vivax merozoite surface protein 1 in individuals exposed to malaria**. *Infect Immun* 1997, **65**(5):1606-1614.
85. Braga EM, Fontes CJ, Krettli AU: **Persistence of humoral response against sporozoite and blood-stage malaria antigens 7 years after a brief exposure to Plasmodium vivax**. *J Infect Dis* 1998, **177**(4):1132-1135.
86. Ceravolo IP, Sanchez BA, Sousa TN, Guerra BM, Soares IS, Braga EM, McHenry AM, Adams JH, Brito CF, Carvalho LH: **Naturally acquired inhibitory antibodies to Plasmodium vivax Duffy binding protein are short-lived and allele-specific following a single malaria infection**. *Clin Exp Immunol* 2009, **156**(3):502-510.
87. Mourao LC, Morais CG, Bueno LL, Jimenez MC, Soares IS, Fontes CJ, Guimaraes Lacerda MV, Xavier MS, Barnwell JW, Galinski MR, Braga EM: **Naturally acquired antibodies to Plasmodium vivax blood-stage vaccine candidates (PvMSP-1-19 and PvMSP-3alpha359-798 and their relationship with hematological features in malaria patients from the Brazilian Amazon**. *Microbes Infect* 2012, **14**(9):730-739.
88. Tobie JE, Abele DC, Hill GJ, 2nd, Contacos PG, Evans CB: **Fluorescent antibody studies on the immune response in sporozoite-induced and blood-induced vivax**

malaria and the relationship of antibody production to parasitemia. *Am J Trop Med Hyg* 1966, **15**(5):676-683.

89. Tobie JE, Abele DC, Wolff SM, Contacos PG, Evans CB: **Serum immunoglobulin levels in human malaria and their relationship to antibody production**. *J Immunol* 1966, **97**(4):498-505.
90. Yeom JS, Kim ES, Lim KJ, Oh JH, Sohn MJ, Yoo SB, Kim E, Bae I, Jung YJ, Park JW: **Naturally acquired IgM antibody response to the C-terminal region of the merozoite surface protein 1 of Plasmodium vivax in Korea: use for serodiagnosis of vivax malaria**. *J Parasitol* 2008, **94**(6):1410-1414.
91. Lim KJ, Park JW, Yeom JS, Lee YH, Yoo SB, Oh JH, Sohn MJ, Bahk YY, Kim YS: **Humoral responses against the C-terminal region of merozoite surface protein 1 can be remembered for more than 30 years in persons exposed to Plasmodium vivax**. *Parasitol Res* 2004, **92**(5):384-389.
92. Park JW, Moon SH, Yeom JS, Lim KJ, Sohn MJ, Jung WC, Cho YJ, Jeon KW, Ju W, Ki CS, Oh MD, Choe K: **Naturally acquired antibody responses to the C-terminal region of merozoite surface protein 1 of Plasmodium vivax in Korea**. *Clin Diagn Lab Immunol* 2001, **8**(1):14-20.
93. Holm I, Nato F, Mendis KN, Longacre S: **Characterization of C-terminal merozoite surface protein-1 baculovirus recombinant proteins from Plasmodium vivax and Plasmodium cynomolgi as recognized by the natural anti-parasite immune response**. *Mol Biochem Parasitol* 1997, **89**(2):313-319.
94. Grimberg BT, Udomsangpetch R, Xainli J, McHenry A, Panichakul T, Sattabongkot J, Cui L, Bockarie M, Chitnis C, Adams J, Zimmerman PA, King CL: **Plasmodium vivax invasion of human erythrocytes inhibited by antibodies directed against the Duffy binding protein**. *PLoS Med* 2007, **4**(12):e337.
95. Barbedo MB, Ricci R, Jimenez MC, Cunha MG, Yazdani SS, Chitnis CE, Rodrigues MM, Soares IS: **Comparative recognition by human IgG antibodies of recombinant proteins representing three asexual erythrocytic stage vaccine candidates of Plasmodium vivax**. *Mem Inst Oswaldo Cruz* 2007, **102**(3):335-339.
96. Herrera S, Bonelo A, Perlaza BL, Valencia AZ, Cifuentes C, Hurtado S, Quintero G, Lopez JA, Corradin G, Arevalo-Herrera M: **Use of long synthetic peptides to study the antigenicity and immunogenicity of the Plasmodium vivax circumsporozoite protein**. *Int J Parasitol* 2004, **34**(13-14):1535-1546.
97. Levitus G, Mertens F, Speranca MA, Camargo LM, Ferreira MU, del Portillo HA: **Characterization of naturally acquired human IgG responses against the N-terminal region of the merozoite surface protein 1 of Plasmodium vivax**. *Am J Trop Med Hyg* 1994, **51**(1):68-76.
98. Oliveira TR, Fernandez-Becerra C, Jimenez MC, Del Portillo HA, Soares IS: **Evaluation of the acquired immune responses to Plasmodium vivax VIR variant antigens in individuals living in malaria-endemic areas of Brazil**. *Malar J* 2006, **5**:83.
99. Pitabut N, Panichakorn J, Mahakunkijcharoen Y, Hirunpetcharat C, Looareesuwan S, Khusmith S: **IgG antibody profile to c-terminal region of Plasmodium vivax merozoite surface protein-1 in Thai individuals exposed to malaria**. *Southeast Asian J Trop Med Public Health* 2007, **38**(1):1-7.
100. Rodrigues MH, Rodrigues KM, Oliveira TR, Comodo AN, Rodrigues MM, Kocken CH, Thomas AW, Soares IS: **Antibody response of naturally infected individuals to recombinant Plasmodium vivax apical membrane antigen-1**. *Int J Parasitol* 2005, **35**(2):185-192.

101. Tapchaisri P, Asavanich A, Limsuwan S, Tharavanij S, Harinasuta KT: **Antibodies against malaria sporozoites in patients with acute uncomplicated malaria and patients with cerebral malaria**. *Am J Trop Med Hyg* 1985, **34**(5):831-836.
102. Wickramarachchi T, Illeperuma RJ, Perera L, Bandara S, Holm I, Longacre S, Handunnetti SM, Udagama-Randeniya PV: **Comparison of naturally acquired antibody responses against the C-terminal processing products of Plasmodium vivax Merozoite Surface Protein-1 under low transmission and unstable malaria conditions in Sri Lanka**. *Int J Parasitol* 2007, **37**(2):199-208.
103. Zakeri S, Babaeekhou L, Mehrizi AA, Abbasi M, Djadid ND: **Antibody responses and avidity of naturally acquired anti-Plasmodium vivax Duffy binding protein (PvDBP) antibodies in individuals from an area with unstable malaria transmission**. *Am J Trop Med Hyg* 2011, **84**(6):944-950.
104. Kim TS, Kim HH, Lee SS, Na BK, Lin K, Cho SH, Kang YJ, Kim DK, Sohn Y, Kim H *et al*: **Prevalence of Plasmodium vivax VK210 and VK247 subtype in Myanmar**. *Malar J* 2010, **9**:195.
105. Storti-Melo LM, da Costa DR, Souza-Neiras WC, Cassiano GC, Couto V, Povoa MM, Soares ID, de Carvalho LH, Arevalo-Herrera M, Herrera S, Rossit AR, Cordeiro JA, de Mattos LC, Machado RL: **Influence of HLA-DRB-1 alleles on the production of antibody against CSP, MSP-1, AMA-1, and DBP in Brazilian individuals naturally infected with Plasmodium vivax**. *Acta Trop* 2012, **121**(2):152-155.
106. Sungkapong T, Culleton R, Yahata K, Tachibana M, Ruengveerayuth R, Udomsangpetch R, Torii M, Tsuboi T, Sattabongkot J, Kaneko O, Chotivanich K: **Humoral immune responses to Plasmodium vivax subtelomeric transmembrane proteins in Thailand**. *Southeast Asian J Trop Med Public Health* 2011, **42**(6):1313-1321.
107. Fernandez-Becerra C, Pein O, de Oliveira TR, Yamamoto MM, Cassola AC, Rocha C, Soares IS, de Braganca Pereira CA, del Portillo HA: **Variant proteins of Plasmodium vivax are not clonally expressed in natural infections**. *Mol Microbiol* 2005, **58**(3):648-658.
108. Kim TS, Kim HH, Kim JY, Kong Y, Na BK, Lin K, Moon SU, Kim YJ, Kwon MH, Sohn Y, Kim H, Lee HW: **Comparison of the antibody responses to Plasmodium vivax and Plasmodium falciparum antigens in residents of Mandalay, Myanmar**. *Malar J* 2011, **10**:228.
109. Yildiz Zeyrek F, Babaoglu A, Demirel S, Erdogan DD, Ak M, Korkmaz M, Coban C: **Analysis of naturally acquired antibody responses to the 19-kd C-terminal region of merozoite surface protein-1 of Plasmodium vivax from individuals in Sanliurfa, Turkey**. *Am J Trop Med Hyg* 2008, **78**(5):729-732.
110. Cui L, Escalante AA, Imwong M, Snounou G: **The genetic diversity of Plasmodium vivax populations**. *Trends Parasitol* 2003, **19**(5):220-226.
111. Cui L, Mascorro CN, Fan Q, Rzomp KA, Khuntirat B, Zhou G, Chen H, Yan G, Sattabongkot J: **Genetic Diversity and the Multiple Infections of *Plasmodium vivax* malaria in Western Thailand**. *Am J Trop Med Hyg* 2003, **68**(5):613-619.
112. Mendis KN, David PH, Carter R: **Human immune responses against sexual stages of malaria parasites: considerations for malaria vaccines** *Int J Parasitol* 1990, **20**(4):497-502.
113. McCarthy VC, Clyde DF: **Plasmodium vivax: Correlation of circumsporozoite precipitation (CSP) reaction with sporozoite-lnduced protective immunity in man**. *Exp Parasitol* 1977, **41**(1):167-171.

114. Haghi AM, Khoramizade MR, Nateghpour M, Mohebali M, Edrissian GH, Eshraghian MR, Sepehrizadeh Z: **A recombinant Plasmodium vivax apical membrane antigen-1 to detect human infection in Iran**. *Korean J Parasitol* 2012, **50**(1):15-21.
115. Chen JH, Wang Y, Ha KS, Lu F, Suh IB, Lim CS, Park JH, Takeo S, Tsuboi T, Han ET: **Measurement of naturally acquired humoral immune responses against the C-terminal region of the Plasmodium vivax MSP1 protein using protein arrays**. *Parasitol Res* 2011, **109**(5):1259-1266.
116. Rodrigues MHC, Cunha MG, Machado RLD, Ferreira Jr OC, Rodrigues MM, Soares IS: **Serological detection of Plasmodium vivax malaria using recombinant proteins corresponding to the 19-kDa C-terminal region of the merozoite surface protein-1**. *Malar J* 2003, **2**:1-7.
117. Garg S, Chauhan SS, Singh N, Sharma YD: **Immunological responses to a 39.8kDa Plasmodium vivax tryptophan-rich antigen (PvTRAg39.8) among humans**. *Microbes Infect* 2008, **10**(10-11):1097-1105.
118. Wickramarachchi T, Premaratne PH, Perera KLRL, Bandara S, Kocken CHM, Thomas AW, Handunnetti SM, Udagama-Randeniya PV: **Natural Human Antibody Responses to Plasmodium vivax Apical Membrane Antigen 1 under Low Transmission and Unstable Malaria Conditions in Sri Lanka**. *Infect Immun* 2006, **74**(1):798-801.
119. Mehrizi AA, Zakeri S, Salmanian AH, Sanati MH, Djadid ND: **IgG subclasses pattern and high-avidity antibody to the C-terminal region of merozoite surface protein 1 of Plasmodium vivax in an unstable hypoendemic region in Iran**. *Acta Trop* 2009, **112**(1):1-7.
120. del Portillo HA, Levitus G, Camargo LM, Ferreira MU, Mertens F: **Human IgG responses against the N-terminal region of the Merozoite Surface Protein 1 of Plasmodium vivax**. *Mem Inst Oswaldo Cruz* 1992, **87 Suppl 3**:77-84.
121. Zeeshan M, Bora H, Sharma YD: **Presence of memory T cells and naturally acquired antibodies in Plasmodium vivax malaria-exposed individuals against a group of tryptophan-rich antigens with conserved sequences**. *J Infect Dis* 2013, **207**(1):175-185.
122. Arruda ME, Zimmerman RH, Souza RM, Oliveira-Ferreira J: **Prevalence and level of antibodies to the circumsporozoite protein of human malaria parasites in five states of the Amazon region of Brazil**. *Mem Inst Oswaldo Cruz* 2007, **102**(3):367-371.
123. Bastos MS, da Silva-Nunes M, Malafronte RS, Hoffmann EH, Wunderlich G, Moraes SL, Ferreira MU: **Antigenic polymorphism and naturally acquired antibodies to Plasmodium vivax merozoite surface protein 1 in rural Amazonians**. *Clin Vaccine Immunol* 2007, **14**(10):1249-1259.
124. Burkot TR, Graves PM, Wirtz RA, Brabin BJ, Battistutta D, Cattani JA, Maizels RM, Alpers MP: **Differential antibody responses to Plasmodium falciparum and P. vivax circumsporozoite proteins in a human population**. *J Clin Microbiol* 1989, **27**(6):1346-1351.
125. Cook J, Speybroeck N, Sochanta T, Somony H, Sokny M, Claes F, Lemmens K, Theisen M, Soares IS, D'Alessandro U, Coosemans M, Erhart A: **Sero-epidemiological evaluation of changes in Plasmodium falciparum and Plasmodium vivax transmission patterns over the rainy season in Cambodia**. *Malar J* 2012, **11**:86.
126. De Arruda M, Souza RC, Veiga ME, Ferreira AF, Zimmerman RH: **Prevalence of Plasmodium vivax variants VK247 and P. vivax-like human malaria: A**

retrospective study in indigenous Indian populations of the Amazon region of Brazil. *Trans R Soc Trop Med Hyg* 1998, **92**(6):628.

127. Del Giudice G, Lambert PH, Mendis K, Pessi A, Tanner M: **Antibody-Responses to Plasmodium-Falciparum and Plasmodium-Vivax Sporozoites in Areas with Stable and Unstable Malaria**. *Bull World Health Organ* 1990, **68**:191-196.
128. Franke ED, Lucas CM, San Roman E: **Antibody response of humans to the circumsporozoite protein of Plasmodium vivax**. *Infect Immun* 1991, **59**(8):2836-2838.
129. Fraser T, Michon P, Barnwell JW, Noe AR, Al-Yaman F, Kaslow DC, Adams JH: **Expression and serologic activity of a soluble recombinant Plasmodium vivax Duffy binding protein**. *Infect Immun* 1997, **65**(7):2772-2777.
130. Gordon DM, Davis DR, Lee M, Lambros C, Harrison BA, Samuel R, Campbell GH, Jegathesan M, Selvarajan K, Lewis GE, Jr.: **Significance of circumsporozoite-specific antibody in the natural transmission of Plasmodium falciparum, Plasmodium vivax, and Plasmodium malariae in an aboriginal (Orang Asli) population of central peninsula Malaysia**. *Am J Trop Med Hyg* 1991, **45**(1):49-56.
131. Herrera S, Gomez A, Vera O, Vergara J, Valderrama-Aguirre A, Maestre A, Mendez F, Wang R, Chitnis CE, Yazdani SS, Arévalo-Herrera M: **Antibody response to Plasmodium vivax antigens in Fy-negative individuals from the Colombian Pacific coast**. *Am J Trop Med Hyg* 2005, **73**(5 Suppl):44-49.
132. Kano FS, Sanchez BA, Sousa TN, Tang ML, Saliba J, Oliveira FM, Nogueira PA, Goncalves AQ, Fontes CJ, Soares IS, Brito CF, Rocha RS, Carvalho LH: **Plasmodium vivax Duffy binding protein: baseline antibody responses and parasite polymorphisms in a well-consolidated settlement of the Amazon Region**. *Trop Med Int Health* 2012, **17**(8):989-1000.
133. Kremsner PG, Neifer S, Zotter GM, Bienzle U, Rocha RM, Maracic M, Clavijo P, Nussenzweig RS, Cochrane AH: **Prevalence and Level of Antibodies to the Circumsporozoite Proteins of Human Malaria Parasites, Including a Variant of Plasmodium-Vivax, in the Population of 2 Epidemiologically Distinct Areas in the State of Acre, Brazil**. *Trans R Soc Trop Med Hyg* 1992, **86**(1):23-27.
134. Lee M, Davis DR, Ballou WR, Folena-Wasserman G, Lewis GE: **Interaction of Malaysian sera with Plasmodium vivax sporozoite antigen**. *Am J Trop Med Hyg* 1988, **39**(6):535-539.
135. Lim CS, Yoon JK, Chang EA, Suh IB, An SS, Lee KH, Chung JT, Tockgo YC: **Seroprevalence to the circumsporozoite protein peptide antigen of Plasmodium vivax in Korean children**. *Microbiol Immunol* 2005, **49**(6):521-527.
136. Lima-Junior JC, Jiang J, Rodrigues-da-Silva RN, Banic DM, Tran TM, Ribeiro RY, Meyer VSE, De-Simone SG, Santos F, Moreno A, Barnwell JW, Galinski MR, Oliveira-Ferreira, J: **B cell epitope mapping and characterization of naturally acquired antibodies to the Plasmodium vivax Merozoite Surface Protein-3α (PvMSP-3 α) in malaria exposed individuals from Brazilian Amazon**. *Vaccine* 2011, **29**(9):1801-1811.
137. Mendis C, Del Giudice G, Gamage-Mendis AC, Tougne C, Pessi A, Weerasinghe S, Carter R, Mendis KN: **Anti-circumsporozoite protein antibodies measure age related exposure to malaria in Kataragama, Sri Lanka**. *Parasite Immunol* 1992, **14**(1):75-86.
138. Michon PA, Arevalo-Herrera M, Fraser T, Herrera S, Adams JH: **Serologic responses to recombinant Plasmodium vivax Duffy binding protein in a Colombian village**. *Am J Trop Med Hyg* 1998, **59**(4):597-599.

139. Migot F, Millet P, Chougnet C, Lepers JP, Deloron P: **Humoral and cellular immune responses to the circumsporozoite protein of Plasmodium vivax in Madagascar**. *Am J Trop Med Hyg* 1993, **48**(4):524-529.
140. Nogueira PA, Alves FP, Fernandez-Becerra C, Pein O, Santos NR, Pereira da Silva LH, Camargo EP, del Portillo HA: **A reduced risk of infection with Plasmodium vivax and clinical protection against malaria are associated with antibodies against the N terminus but not the C terminus of merozoite surface protein 1**. *Infect Immun* 2006, **74**(5):2726-2733.
141. Ramasamy R, Nagendran K, Ramasamy MS: **Antibodies to epitopes on merozoite and sporozoite surface antigens as serologic markers of malaria transmission: studies at a site in the dry zone of Sri Lanka**. *Am J Trop Med Hyg* 1994, **50**(5):537-547.
142. Ramasamy R, Nagendran K, Ramasamy MS: **Dynamics of natural antibody responses to malaria parasite surface proteins in the intermediate rainfall zone of Sri Lanka**. *Indian J Med Res* 1995, **101**:66-74.
143. Seth RK, Bhat AA, Rao DN, Biswas S: **Acquired immune response to defined Plasmodium vivax antigens in individuals residing in northern India**. *Microbes Infect* 2010, **12**(3):199-206.
144. Tran TM, Oliveira-Ferreira J, Moreno A, Santos F, Yazdani SS, Chitnis CE, Altman JD, Meyer EV, Barnwell JW, Galinski MR: **Comparison of IgG reactivities to Plasmodium vivax merozoite invasion antigens in a Brazilian Amazon population**. *Am J Trop Med Hyg* 2005, **73**(2):244-255.
145. Van den Eede P, Soto-Calle VE, Delgado C, Gamboa D, Grande T, Rodriguez H, Llanos-Cuentas A, Anne J, D'Alessandro U, Erhart A: **Plasmodium vivax sub-patent infections after radical treatment are common in Peruvian patients: results of a 1-year prospective cohort study**. *PloS One* 2011, **6**(1):e16257.
146. Xainli J, Cole-Tobian JL, Baisor M, Kastens W, Bockarie M, Yazdani SS, Chitnis CE, Adams JH, King CL: **Epitope-specific humoral immunity to Plasmodium vivax Duffy binding protein**. *Infect Immun* 2003, **71**(5):2508-2515.
147. Yildiz Zeyrek F, Palacpac N, Yuksel F, Yagi M, Honjo K, Fujita Y, Arisue N, Takeo S, Tanabe K, Horii T, Tsuboi T, Ishii KJ, Coban C: **Serologic markers in relation to parasite exposure history help to estimate transmission dynamics of Plasmodium vivax**. *PloS One* 2011, **6**(11):e28126.
148. Soares IS, Oliveira SG, Souza JM, Rodrigues MM: **Antibody response to the N and C-terminal regions of the Plasmodium vivax Merozoite Surface Protein 1 in individuals living in an area of exclusive transmission of P. vivax malaria in the north of Brazil**. *Acta Trop* 1999, **72**(1):13-24.
149. Lima-Junior JC, Rodrigues-da-Silva RN, Banic DM, Jiang J, Singh B, Fabricio-Silva GM, Porto LC, Meyer EV, Moreno A, Rodrigues MM, Barnwell JW, Galinski MR, de Oliveira-Ferreira, J: **Influence of HLA-DRB1 and HLA-DQB1 alleles on IgG antibody response to the P. vivax MSP-1, MSP-3alpha and MSP-9 in individuals from Brazilian endemic area**. *PloS One* 2012, **7**(5):e36419.
150. Oliveira-Ferreira J, Pratt-Riccio LR, Arruda M, Santos F, Ribeiro CT, Goldberg AC, Banic DM: **HLA class II and antibody responses to circumsporozoite protein repeats of P. vivax (VK210, VK247 and P. vivax-like) in individuals naturally exposed to malaria**. *Acta Trop* 2004, **92**(1):63-69.
151. Versiani FG, Almeida ME, Melo GC, Versiani FO, Orlandi PP, Mariuba LA, Soares LA, Souza LP, da Silva Balieiro AA, Monteiro WM, Costa FT, del Portillo HA, Lacerda MV, Nogueira PA: **High levels of IgG3 anti ICB2-5 in Plasmodium vivax-infected individuals who did not develop symptoms**. *Malar J* 2013, **12**(1):294.

152. Wongsrichanalai C, Webster HK, Permpanich B, Chuanak N, Ketrangsri S: **Naturally acquired circumsporozoite antibodies and their role in protection in endemic falciparum and vivax malaria**. *Am J Trop Med Hyg* 1991, **44**(2):201-204.
153. Chootong P, Panichakul T, Permmongkol C, Barnes SJ, Udomsangpetch R, Adams JH: **Characterization of inhibitory anti-Duffy binding protein II immunity: approach to Plasmodium vivax vaccine development in Thailand**. *PloS One* 2012, **7**(4):e35769.
154. Fowkes FJ, McGready R, Cross NJ, Hommel M, Simpson JA, Elliott SR, Richards JS, Lackovic K, Viladpai-Nguen J, Narum D, Tsuboi T, Anders RF, Nosten F, Beeson JG: **New insights into acquisition, boosting, and longevity of immunity to malaria in pregnant women**. *J Infect Dis* 2012, **206**(10):1612-1621.
155. Jones TR, Yuan LF, Marwoto HA, Gordon DM, Wirtz RA, Hoffman SL: **Low immunogenicity of a Plasmodium vivax circumsporozoite protein epitope bound by a protective monoclonal antibody**. *Am J Trop Med Hyg* 1992, **47**(6):837-843.
156. Ladeia-Andrade S, Ferreira MU, Scopel KK, Braga EM, Bastos Mda S, Wunderlich G, Coura JR: **Naturally acquired antibodies to merozoite surface protein (MSP)-1(19) and cumulative exposure to Plasmodium falciparum and Plasmodium vivax in remote populations of the Amazon Basin of Brazil**. *Mem Inst Oswaldo Cruz* 2007, **102**(8):943-951.
157. Lima-Junior JC, Tran TM, Meyer EV, Singh B, De-Simone SG, Santos F, Daniel-Ribeiro CT, Moreno A, Barnwell JW, Galinski MR, Oliveira-Ferreira J: **Naturally acquired humoral and cellular immune responses to Plasmodium vivax merozoite surface protein 9 in Northwestern Amazon individuals**. *Vaccine* 2008, **26**(51):6645-6654.
158. Wirtz RA, Rosenberg R, Sattabongkot J, Webster HK: **Prevalence of antibody to heterologous circumsporozoite protein of Plasmodium vivax in Thailand**. *Lancet* 1990, **336**(8715):593-595.
159. Cochrane AH, Nardin EH, de Arruda M, Maracic M, Clavijo P, Collins WE, Nussenzweig RS: **Widespread reactivity of human sera with a variant repeat of the circumsporozoite protein of Plasmodium vivax**. *Am J Trop Med Hyg* 1990, **43**(5):446-451.
160. Pessi A, Michel M, Bianchi E, Mendis A, Tougne C, Verdini AS, Lambert P-H, Carter R, Mendis K, Giudice G: **Use of Synthetic Peptides in the Study of the Antibody Response to Plasmodium Vivax Sporozoites**. *Am J Trop Med Hyg* 1990, **42**(1):17-23.
161. Graves PM, Bhatia K, Burkot TR, Prasad M, Wirtz RA, Beckers P: **Association between HLA type and antibody response to malaria sporozoite and gametocyte epitopes is not evident in immune Papua New Guineans**. *Clin Exp Immunol* 1989, **78**(3):418-423.
162. Molina DM, Finney OC, Arevalo-Herrera M, Herrera S, Felgner PL, Gardner MJ, Liang XW, Wang RB: **Plasmodium vivax Pre-Erythrocytic Stage Antigen Discovery: Exploiting Naturally Acquired Humoral Responses**. *Am J Trop Med Hyg* 2012, **87**(3):460-469.
