# Supplementary material for: Immunological markers of Plasmodium vivax exposure and immunity: a systematic review and meta-analysis
Source: BMC Med. 2014 Sep 9;12:150. doi: 10.1186/s12916-014-0150-1 (PMC4172944; doi:10.1186/s12916-014-0150-1)
Supplement: Additional file 4 — Details of recombinant antigens featured in systematic review. [file 12916_2014_150_MOESM4_ESM.pdf]

Additional file 4: Details of recombinant antigens featured in the systematic review

**Author, year [reference]**	**Allele/Strain (region)**	**Expression System**	**Tag**	**Seroprevalence (%)**
***Pv*CSP**				
Oliveira-Ferreira, 2004 [1]	VK210	synthetic	NA	34.26
Oliveira-Ferreira, 2004 [1]	VK247	synthetic	NA	24.07
Oliveira-Ferreira, 2004 [1]	*P. vivax*-like	synthetic	NA	31.48
Wongsrichanalai, 1991 [2]	VK210 (NS1/81-V20)[1]	DNS	DNS	Controls: 44.8, Cases: 66.7
Yildiz Zeyrek, 2011 [3]	PvCSP-c chimeric protein containing VK210 and VK247 amino acid repeat sequences	Wheat germ cell-free translational system, *E. coli*	His	IgG: 33.8, IgM: 36.9, IgG1: 25.6, IgG2: 2.05, IgG3: 28.7, IgG4: 5.64[2]
***Pv*DBPII**				
Cole-Tobian, 2009 [4]	PNG-AH (251-566)	*E. coli*	His	98
Cole-Tobian, 2009 [4]	PNG-O (251-566)	*E. coli*	His	90
Cole-Tobian, 2009 [4]	PNG-P (251-566)	*E. coli*	His	90
Cole-Tobian, 2009 [4]	Sal 1 (251-566)	*E. coli*	His	72
Tran, 2005 [5]	DNS (194-521)	*E. coli*	His	Ribeirinha: 69, Colina: 70
***Pv*DBPII-IV**				
Kano, 2012 [6]	Sal 1 (132-771)	*E. coli*	GST	49.5
Souza-Silva, 2010 [7]	132-771	*E. coli*	GST	18.6
***Pv*MSP-1$_{19}$**				
Ak, 1998 [8]	DNS (1639-1729)	*S. cerevisiae*	His	IgG + IgM: 70

[1] NS1/81-V20 consists of 81 amino acids from the non-structural protein 1 of influenza A virus fused N-terminally to the entire repeat region of the CS protein of *P. vivax*
[2] Frequencies calculated from 2 x 2 provided by authors

Kano, 2012 [6]	Belem (1616-1704)	*E. coli*	His	60
Lima-Junior, 2012 [9]	Belem (1616-1704)	*E. coli*	His	86.7
Cole-Tobian, 2009 [4]	DNS	*E. coli*	DNS	83.4
Fernandez-Becerra, 2010 [10]	Belem (1615-1726)	*E. coli*	GST	80
Nogueira, 2006 [11]	Belem (1615-1726)	*E. coli*	GST	IgG: 42.8, (IgG1: 25, IgG2: 0, IgG3: 0, IgG4: 0)[3]
Yildiz Zeyrek, 2011 [3]	Sal 1 (1590-1699)	Wheat germ cell-free translational system	His	IgG: 50.3, IgM: 28.2, IgG1: 45.6, IgG2: 5.6, IgG3: 35.4, IgG4: 2.56[4]
***Pv*MSP-1$_{NT}$**				
Versiani, 2013 [12]	Belem (170-675)	*E. coli*	GST	IgG: 35.4, (IgG1: 8.7, IgG2: 29.6, IgG3: 51.6, IgG4: 8.7%)[5]
Nogueira, 2006 [11]	Belem (170-675)	*E. coli*	GST	IgG: 36.4, (IgG1: 17, IgG2: 0, IgG3: 56, IgG4: 0)[6]
Fernandez-Becerra, 2010 [10]	DNS	*E. coli*	GST	89
***Pv*MSP-3α$_{FL}$**				
Lima-Junior, 2011 [13]	Belem (73-2520)	*E. coli*	His	78
***Pv*MSP-3α$_{NT}$**				
Lima-Junior, 2011 [13]	Belem (73-309)	*E. coli*	His	39
Stanisic, 2013 [14]	Belem (73-309)	*E. coli*	His	38.3
***Pv*MSP-3α$_{RI}$**				
Lima-Junior, 2011 [13]	Belem (316-1242)	*E. coli*	His	64
Stanisic, 2013 [14]	Belem (316-1242)	*E. coli*	His	36.1

[3] Frequencies of subclass responses were reported for IgG responders only and were estimated from a graph presented in the original paper.
[4] Frequencies calculated from 2 x 2 provided by authors
[5] Frequencies of subclass responses were reported (in text) for IgG responders only.
[6] Frequencies of subclass responses were reported for IgG responders only and were estimated from a graph presented in the original paper.

***Pv*MSP-3α$_{RII}$**				
Lima-Junior, 2011 [13]	Belem (1246-2058)	*E. coli*	His	53
Stanisic, 2013 [14]	Belem (1246-2058)	*E. coli*	His	38.3
***Pv*MSP-3α$_{CT}$**				
Lima-Junior, 2011 [13]	Belem (2059-2523)	*E. coli*	His	54
Stanisic, 2013 [14]	Belem (2059-2523)	*E. coli*	His	65
***Pv*MSP-5**				
Woodberry, 2008 [15]	Sal 1	*E. coli*	His	IgG: 47, 42; IgM: 46, 49; IgG1: 24, 50; IgG2: 5, 6; IgG3: 85, 69; IgG4: 0, 0 (controls, cases)[7]
***Pv*MSP-9$_{RIRII}$**				
Lima-Junior, 2012 [9]	Belem (729-972)	*E. coli*	His	63.7
Lima-Junior, 2008 [16]	Belem (729-972)	*E. coli*	His	50
Stanisic, 2013 [14]	Belem (729-972)	*E. coli*	GST	8.7
***Pv*MSP-9$_{RII}$**				
Lima-Junior, 2012 [9]	Belem (874-972)	*E. coli*	His	40.2
Lima-Junior, 2008 [16]	Belem (874-972)	*E. coli*	His	40
***Pv*MSP-9$_{NT}$**				
Lima-Junior, 2012 [9]	Belem (34-367)	*E. coli*	His	33.7
Lima-Junior, 2008 [16]	Belem (34-367)	*E. coli*	His	16
Stanisic, 2013 [14]	Belem (34-193)	*E. coli*	GST	45.9
***Pv*AMA1 ectodomain**				
Fowkes, 2012 [17]	Sal 1 (77-484)	Wheat germ cell-free translational	His	Controls: 9, Cases: 38)

[7] Frequencies of subclass responses were reported for IgG responders only.

		system		
Yildiz-Zeyrek, 2011 [3]	Sal 1 (77-484)	Wheat germ cell-free translational system	His	IgG: 21.0, IgM: 18, IgG1: 20, IgG2: 1.54, IgG3: 15.9, IgG4: 1.54[8]
***Pv*RBP1 extracellular domain**				
Tran, 2005 [5]	Belem	*E. coli*	His	Ribeirinha: 73, Colina: 57
***Pv*SERA4**				
Yildiz-Zeyrek, 2011 [3]	Sal 1 (19-352)	*E. coli*	His	IgG: 16.4, IgM: 49.2, IgG1: 14.9, IgG2: 7.69, IgG3: 9.7, IgG4: 2.05[9]

Region shows amino acid positions. If no position is given then BLAST searches were performed with amino acid sequence. Seroprevalence for IgG is shown unless otherwise indicated. Abbreviations: CT, C-terminal; DNS, data not shown in original manuscript; GST, Glutathione *S*-Transferase; His, histadine; MSP, Merozoite Surface Protein; NA, Not Applicable; NT, N-terminal; RI, Block I repeats; RII, Block II repeats; RIRII, Block I and Block II repeats.

8 Frequencies calculated from 2 x 2 provided by authors
[9] Frequencies calculated from 2 x 2 provided by authors

References:

1. Oliveira-Ferreira J, Pratt-Riccio LR, Arruda M, Santos F, Ribeiro CT, Goldberg AC, Banic DM: **HLA class II and antibody responses to circumsporozoite protein repeats of P. vivax (VK210, VK247 and P. vivax-like) in individuals naturally exposed to malaria**. *Acta Trop* 2004, **92**(1):63-69.
2. Wongsrichanalai C, Webster HK, Permpanich B, Chuanak N, Ketrangsri S: **Naturally acquired circumsporozoite antibodies and their role in protection in endemic falciparum and vivax malaria**. *Am J Trop Med Hyg* 1991, **44**(2):201-204.
3. Yildiz Zeyrek F, Palacpac N, Yuksel F, Yagi M, Honjo K, Fujita Y, Arisue N, Takeo S, Tanabe K, Horii T, Tsuboi T, Ishii KJ, Coban C: **Serologic markers in relation to parasite exposure history help to estimate transmission dynamics of Plasmodium vivax**. *PloS One* 2011, **6**(11):e28126.
4. Cole-Tobian JL, Michon P, Biasor M, Richards JS, Beeson JG, Mueller I, King CL: **Strain-specific duffy binding protein antibodies correlate with protection against infection with homologous compared to heterologous plasmodium vivax strains in Papua New Guinean children**. *Infect Immun* 2009, **77**(9):4009-4017.
5. Tran TM, Oliveira-Ferreira J, Moreno A, Santos F, Yazdani SS, Chitnis CE, Altman JD, Meyer EV, Barnwell JW, Galinski MR: **Comparison of IgG reactivities to Plasmodium vivax merozoite invasion antigens in a Brazilian Amazon population**. *Am J Trop Med Hyg* 2005, **73**(2):244-255.
6. Kano FS, Sanchez BA, Sousa TN, Tang ML, Saliba J, Oliveira FM, Nogueira PA, Goncalves AQ, Fontes CJ, Soares IS, Brito CF, Rocha RS, Carvalho LH: **Plasmodium vivax Duffy binding protein: baseline antibody responses and parasite polymorphisms in a well-consolidated settlement of the Amazon Region**. *Trop Med Int Health* 2012, **17**(8):989-1000.
7. Souza-Silva FA, Da Silva-Nunes M, Sanchez BAM, Ceravolo IP, Malafronte RS, Brito CFA, Ferreira MU, Carvalho LH: **Naturally acquired antibodies to Plasmodium vivax Duffy binding protein (DBP) in rural Brazilian Amazon**. *Am J Trop Med Hyg* 2010, **82**(2):185-193.
8. Ak M, Jones TR, Charoenvit Y, Kumar S, Kaslow DC, Maris D, Marwoto H, Masbar S, Hoffman SL: **Humoral immune responses against Plasmodium vivax MSP1 in humans living in a malaria endemic area in flores, Indonesia**. *Southeast Asian J Trop Med Public Health* 1998, **29**(4):685-691.
9. Lima-Junior JC, Rodrigues-da-Silva RN, Banic DM, Jiang J, Singh B, Fabricio-Silva GM, Porto LC, Meyer EV, Moreno A, Rodrigues MM, Barnwell JW, Galinski MR, de Oliveira-Ferreira J: **Influence of HLA-DRB1 and HLA-DQB1 alleles on IgG antibody response to the P. vivax MSP-1, MSP-3alpha and MSP-9 in individuals from Brazilian endemic area**. *PloS One* 2012, **7**(5):e36419.
10. Fernandez-Becerra C, Sanz S, Brucet M, Stanisic DI, Alves FP, Camargo EP, Alonso PL, Mueller I, del Portillo HA: **Naturally-acquired humoral immune responses against the N- and C-termini of the Plasmodium vivax MSP1 protein in endemic regions of Brazil and Papua New Guinea using a multiplex assay**. *Malar J* 2010, **9**:29.
11. Nogueira PA, Alves FP, Fernandez-Becerra C, Pein O, Santos NR, Pereira da Silva LH, Camargo EP, del Portillo HA: **A reduced risk of infection with Plasmodium vivax and clinical protection against malaria are associated with antibodies**

against the N terminus but not the C terminus of merozoite surface protein 1. *Infect Immun* 2006, **74**(5):2726-2733.

12. Versiani FG, Almeida ME, Melo GC, Versiani FO, Orlandi PP, Mariuba LA, Soares LA, Souza LP, da Silva Balieiro AA, Monteiro WM, Costa FT, del Portillo HA, Lacerda MV, Nogueira PA: **High levels of IgG3 anti ICB2-5 in Plasmodium vivax-infected individuals who did not develop symptoms**. *Malar J* 2013, **12**(1):294.
13. Lima-Junior JC, Jiang J, Rodrigues-da-Silva RN, Banic DM, Tran TM, Ribeiro RY, Meyer VSE, De-Simone SG, Santos F, Moreno A, Barnwell JW, Galinski MR, Oliveira-Ferreira J: **B cell epitope mapping and characterization of naturally acquired antibodies to the Plasmodium vivax Merozoite Surface Protein-3α (PvMSP-3 α) in malaria exposed individuals from Brazilian Amazon**. *Vaccine* 2011, **29**(9):1801-1811.
14. Stanisic DI, Javati S, Kiniboro B, Lin E, Jiang J, Singh B, Meyer EV, Siba P, Koepfli C, Felger I, Galinski MR, Mueller, I: **Naturally Acquired Immune Responses to P. vivax Merozoite Surface Protein 3alpha and Merozoite Surface Protein 9 Are Associated with Reduced Risk of P. vivax Malaria in Young Papua New Guinean Children**. *PLoS Negl Trop Dis* 2013, **7**(11):e2498.
15. Woodberry T, Minigo G, Piera KA, Hanley JC, de Silva HD, Salwati E, Kenangalem E, Tjitra E, Coppel RL, Price RN, Anstey NM, Plebanski M: **Antibodies to Plasmodium falciparum and Plasmodium vivax merozoite surface protein 5 in Indonesia: species-specific and cross-reactive responses**. *J Infect Dis* 2008, **198**(1):134-142.
16. Lima-Junior JC, Tran TM, Meyer EV, Singh B, De-Simone SG, Santos F, Daniel-Ribeiro CT, Moreno A, Barnwell JW, Galinski MR, Oliveira-Ferreira J: **Naturally acquired humoral and cellular immune responses to Plasmodium vivax merozoite surface protein 9 in Northwestern Amazon individuals**. *Vaccine* 2008, **26**(51):6645-6654.
17. Fowkes FJ, McGready R, Cross NJ, Hommel M, Simpson JA, Elliott SR, Richards JS, Lackovic K, Viladpai-Nguen J, Narum D, Tsuboi T, Anders RF, Nosten F, Beeson JG: **New insights into acquisition, boosting, and longevity of immunity to malaria in pregnant women**. *J Infect Dis* 2012, **206**(10):1612-1621.
