# Supplementary material for: Immunological markers of Plasmodium vivax exposure and immunity: a systematic review and meta-analysis
Source: BMC Med. 2014 Sep 9;12:150. doi: 10.1186/s12916-014-0150-1 (PMC4172944; doi:10.1186/s12916-014-0150-1)
Supplement: Additional file 5 — IgG Subclass analysis. [file 12916_2014_150_MOESM5_ESM.docx]

**Additional file 5: IgG subclass analysis**

## IgG subclass responses to PvCSP repeat region

IgG subclass responses to *Pv*CSP (VK210 and VK247 combined alleles) were explored in the cross-sectional study by Yidiz-Zeyrek *et al* [[1](#_ENREF_1)]. The predominant IgG subclasses were IgG1 and IgG3, but all 4 subclasses were associated with increased odds of *P. vivax* infection with varying degrees of significance: IgG1 (OR 1.83, 95% CI 0.95-3.49), IgG2 (OR 1.45, 95% CI 0.20-10.5), IgG3 (OR 2.12, 95% CI 1.13-3.98), IgG4 (OR 1.21, 95% CI 0.36-4.11) [[1](#_ENREF_1)].

***IgG subclass responses to PvMSP-1_19_***

IgG1 and IgG3 were the predominant subclasses to *Pv*MSP-1_19_ in two studies and showed similar patterns to that observed for total IgG (**Additional file 3** and **Figure 3**) [[1](#_ENREF_1), [2](#_ENREF_2)]. All IgG subclass responses were associated with increased odds of *P. vivax* infection: IgG1 (OR 5.24, 95% CI 2.82-9.74), IgG2 (OR 7.16, 95% CI 1.50-34.1), IgG3 (OR 7.51, 95% CI 3.88-14.52) and IgG4 (OR 2.20, 95% CI 0.36-13.48) [[1](#_ENREF_1)].

***IgG subclass responses to PvMSP-1_NT_***

The study by Versiani *et al* showed that whilst IgG2 (RR 0.29, 95% CI 0.04-2.06) and IgG3 (RR 0.50, 95% CI 0.16-1.54) subclass responses to *Pv*MSP-1_NT_ were associated with decreased risk of *P. vivax* infection, IgG1 (RR 7.43, 95% CI 5.26-10.5) and IgG4 (RR 7.43, 95% CI 5.26-10.49) responses were associated with increased risk of *P. vivax* infection [[3](#_ENREF_3)]. In this study IgG3 was the most prevalent subclass response to *Pv*MSP-1_NT_, followed by IgG2 (**Additional file 3**) [[3](#_ENREF_3)]. In contrast, Nogueira showed that among IgG responders to *Pv*MSP-1_NT_, IgG3 was the most prevalent subclass response, followed by IgG1 (**Additional file 3**) [[4](#_ENREF_4)].

***IgG subclass responses to*** *Pv****MSP-5***

Analysis of IgG subclass responses to *Pv*MSP-5 was performed by Woodberry *et al*. IgG responses were predominantly IgG3 followed by IgG1 for *Pv*MSP5 and associations reflected those observed for IgG (**Additional file 3**)[[5](#_ENREF_5)]. No association was shown between IgG1 (OR 1.77, 95% CI 0.76-4.12), IgG2 (OR 1.01, 95% CI 0.14-7.35) or IgG3 (OR 0.61, 95% CI 0.32-1.15) responses and odds of symptomatic *P. vivax* [[5](#_ENREF_5)].

***IgG subclass responses to*** *Pv****AMA1 ectodomain***

A cross-sectional study investigating the association between anti-*Pv*AMA1 ectodomain responses and prevalence of *P. vivax* infection showed that IgG1 responses (OR 3.57, 95% CI 1.69-7.52) were associated with increased odds of *P. vivax* infection, suggesting that antibodies to this antigen may represent a marker for exposure [[1](#_ENREF_1)]. There was no evidence that IgG2 (OR 2.92, 95% CI 0.26-32.8), IgG3 (OR 1.95, 95% CI 0.90-4.22), or IgG4 (OR 0.72, 95% CI 0.06-8.02) responses were associated with odds of *P. vivax* infection [[1](#_ENREF_1)].

***IgG subclass responses to PvSERA4***

A single cross-sectional study investigating the association between anti-*Pv*SERA4 responses and *P. vivax* infection showed that IgM responders had higher odds of *P. vivax* detected by light microscopy compared with non-responders (OR 2.50, 95% CI 1.39-4.49) [[1](#_ENREF_1)]. Similarly, total IgG, IgG1, IgG2 and IgG3 responders tended to have higher odds of *P. vivax* infection compared to non-responders [[1](#_ENREF_1)]. Among IgG responders to *Pv*SERA4, IgG1 was the most prevalent subclass, followed by IgG3 [[1](#_ENREF_1)].

**References:**

1. Yildiz Zeyrek F, Palacpac N, Yuksel F, Yagi M, Honjo K, Fujita Y, Arisue N, Takeo S, Tanabe K, Horii T, Tsuboi T, Ishii KJ, Coban C: **Serologic markers in relation to parasite exposure history help to estimate transmission dynamics of Plasmodium vivax**. *PloS One* 2011, **6**(11):e28126.

2. Yildiz Zeyrek F, Babaoglu A, Demirel S, Erdogan DD, Ak M, Korkmaz M, Coban C: **Analysis of naturally acquired antibody responses to the 19-kd C-terminal region of merozoite surface protein-1 of Plasmodium vivax from individuals in Sanliurfa, Turkey**. *Am J Trop Med Hyg* 2008, **78**(5):729-732.

3. Versiani FG, Almeida ME, Melo GC, Versiani FO, Orlandi PP, Mariuba LA, Soares LA, Souza LP, da Silva Balieiro AA, Monteiro WM, Costa FT, del Portillo HA, Lacerda MV, Nogueira PA: **High levels of IgG3 anti ICB2-5 in Plasmodium vivax-infected individuals who did not develop symptoms**. *Malar J* 2013, **12**(1):294.

4. Nogueira PA, Alves FP, Fernandez-Becerra C, Pein O, Santos NR, Pereira da Silva LH, Camargo EP, del Portillo HA: **A reduced risk of infection with Plasmodium vivax and clinical protection against malaria are associated with antibodies against the N terminus but not the C terminus of merozoite surface protein 1**. *Infect Immun* 2006, **74**(5):2726-2733.

5. Woodberry T, Minigo G, Piera KA, Hanley JC, de Silva HD, Salwati E, Kenangalem E, Tjitra E, Coppel RL, Price RN, Anstey NM, Plebanski M: **Antibodies to Plasmodium falciparum and Plasmodium vivax merozoite surface protein 5 in Indonesia: species-specific and cross-reactive responses**. *J Infect Dis* 2008, **198**(1):134-142.
